# Ribosomal DNA copy number loss and sequence variation in cancer

**DOI:** 10.1371/journal.pgen.1006771

**Published:** 2017-06-22

**Authors:** Baoshan Xu, Hua Li, John M. Perry, Vijay Pratap Singh, Jay Unruh, Zulin Yu, Musinu Zakari, William McDowell, Linheng Li, Jennifer L. Gerton

**Affiliations:** 1 Guangdong Provincial Key Laboratory of Stomatology, Guanghua School of Stomatology, Hospital of Stomatology, Institute of Stomatological Research, Sun Yat-sen University, Guangzhou, Guangdong, China; 2 The Stowers Institute for Medical Research, Kansas City, Missouri, United States of America; 3 University of Kansas Cancer Center, Kansas City, Kansas, United States of America; 4 University of Kansas School of Medicine, Department of Pathology and Laboratory Medicine, Kansas City, Kansas, United States of America; 5 University of Kansas School of Medicine, Department of Biochemistry and Molecular Biology, Kansas City, Kansas, United States of America; Cleveland Clinic Genomic Medicine Institute, UNITED STATES

## Abstract

Ribosomal DNA is one of the most variable regions in the human genome with respect to copy number. Despite the importance of rDNA for cellular function, we know virtually nothing about what governs its copy number, stability, and sequence in the mammalian genome due to challenges associated with mapping and analysis. We applied computational and droplet digital PCR approaches to measure rDNA copy number in normal and cancer states in human and mouse genomes. We find that copy number and sequence can change in cancer genomes. Counterintuitively, human cancer genomes show a **loss** of copies, accompanied by global copy number co-variation. The sequence can also be more variable in the cancer genome. Cancer genomes with lower copies have mutational evidence of mTOR hyperactivity. The PTEN phosphatase is a tumor suppressor that is critical for genome stability and a negative regulator of the mTOR kinase pathway. Surprisingly, but consistent with the human cancer genomes, hematopoietic cancer stem cells from a *Pten*^-/-^ mouse model for leukemia have **lower** rDNA copy number than normal tissue, despite increased proliferation, rRNA production, and protein synthesis. Loss of copies occurs early and is associated with hypersensitivity to DNA damage. Therefore, copy loss is a recurrent feature in cancers associated with mTOR activation. Ribosomal DNA copy number may be a simple and useful indicator of whether a cancer will be sensitive to DNA damaging treatments.

## Author summary

The ribosomal DNA encodes the RNAs needed to make ribosomes for protein synthesis and cellular proliferation. However, ribosomal DNA has been excluded from most mammalian genome-wide studies due to challenges associated with its analysis. We find that both the sequence and copy number of the ribosomal DNA can change in human cancer genomes. mTOR is a kinase that senses the nutritional environment and is often over-active in cancer. Given mutational evidence for mTOR activation in the human cancer genomes with loss of ribosomal DNA copies, we analyzed ribosomal DNA in hematopoietic stem cells derived from mice under conditions of mTOR activation. Like the human cancer genomes, the ribosomal DNA copy number contracts in mTOR activated hematopoietic stem cells relative to normal stem cells. Loss is associated with high rates of cellular proliferation, rRNA production, and protein synthesis, but compromised survival in the presence of DNA damage. Contractions are a recurrent feature in cancer genomes with overactive mTOR and may predict sensitivity to DNA damaging chemotherapeutics. Ribosomal DNA may be altered in other disease contexts.

## Introduction

Repetitive regions in the human genome include ribosomal DNA (rDNA), telomeres, and centromeres, and these regions have profoundly important functions for genome stability [1,2,3]. While challenging to study, genetic variability of these regions is ripe for selection. The rDNA encodes the functional RNAs of the ribosome, the most conserved and utilized genes in the genome. Three of the four RNAs are transcribed initially as a single transcript by RNA polymerase I. The repeats encoding the RNAs are present on five different regions on human acrocentric chromosomes, with each region composed of several copies of the gene, referred to as 45S. 45S is processed into three separate RNAs (28S, 5.8S, and 18S). The fourth RNA is encoded by the 5S genes, located on human chromosome 1 and transcribed by RNA polymerase III. The number of 5S copies correlates with 45S [4]. The loci that encode the rRNAs are transcribed at incredibly high rates, with rRNA often constituting as much as 80% of the RNA in actively proliferating cells, to meet the demand for ribosome production. Even so, only about 50% of the repeats are transcribed.

The number of repeats is variable in human genomes [5], with one recent computational study estimating between 14–410 copies of 45S [4]. The variability in number combined with five different chromosomal locations makes the 45S pattern unique in each human genome. The human 45S genes are highly recombinogenic, with an estimated 10% recombination in a single generation [6]. The sequence of the 45S repeat within a species is highly conserved due presumably to high rates of recombination which allow for concerted evolution [7]. While repeats exist mainly in a head to tail tandem arrangement, palindromic arrangements have also been observed, especially in syndromes with increased genomic instability [8].

One can imagine that one way for a cell to achieve high rates of protein synthesis would be to expand the number of rDNA copies, especially given that ribosomal RNA can be limiting for ribosome biogenesis [9]. In fact, increases in copy number occur in different developmental and disease contexts. Expansions occur in frog oocytes [10] and the macronucleus of tetrahymena [11] as part of the normal course of development, presumably to help achieve high rates of ribosome biogenesis and protein synthesis. Southern blotting experiments demonstrate that recombination at the rDNA is common in adult lung and colorectal cancer [12], suggesting the possibility that the rDNA may be affected in the cancer genome. However, copy number of the rDNA in mammalian development and disease has not been examined.

The TOR (target of rapamycin) kinase detects the nutritional environment and promotes ribosome biogenesis when amino acids are plentiful. The PTEN (phosphate and tensin homolog) phosphatase is a negative regulator of mTOR activity and acts as a tumor suppressor. Somewhat paradoxically, overfeeding flies, which overstimulates mTOR, has been associated with contraction of the rDNA copy number in the germline in *Drosophila*, transmitted transgenerationally [13]. TOR signaling was required for the amplification from low copy number back to normal copy number in budding yeast [14]. Together these results suggest there may be a connection between TOR signaling and the rDNA copy number, but this has not been explored in the mammalian genome. Furthermore, if the copy number or sequence changes in response to the environment, then rDNA may act as both a sensor and adaptor under stress conditions.

In addition to its role in ribosome biogenesis, the rDNA has many extra-ribosome functions, such as regulating gene expression, chromosome organization, and titrating chromatin factors [15,16,17,18]. The nucleolus, which contains the ribosomal DNA, is a hub for many signaling pathways and can act as a stress sensor. Chromosomal domains from most human chromosomes are associated with the nucleolus [19,20], highlighting its role as an organizer. Dramatically reducing the copy number in yeast does not cause reduction in rRNA levels, but causes all repeats to become active (sometimes termed “compensation”) and the yeast are hypersensitive to DNA damage [21]. In contrast, low copy compromises protein synthesis and development in the bobbed mutants in *Drosophila* [22]. Furthermore, copy number of the rDNA titrates position effect variegation in flies [15,18,23]. Therefore, modulation of copy number has the potential to regulate signaling, genome stability, chromosome organization, and gene expression without necessarily affecting ribosome biogenesis.

Recent technological developments enable accurate and high throughput measurements of the copy number of the rDNA. Copy number can be estimated from whole-genome sequence (WGS) and droplet digital PCR (ddPCR). The plethora of cancer genome sequencing projects allows comparison between the copy number in the tumor genome versus the normal genome from a single individual. We used these new technologies to analyze how rDNA copy number changes in different tissues and in cancer. Surprisingly, rDNA copy number is reduced in cancer genomes with mTOR activation, providing evidence that copy number can be altered in a natural system. We also discovered co-occurring increased copy number of additional genes, and evidence for single nucleotide variation in the repeats. Using cancer stem cells derived from a mouse model for leukemia driven by loss of *Pten*, we find that copy loss does not compromise proliferation, rRNA production, or protein synthesis, but cells are hypersensitive to DNA damage. These data suggest that the ribosomal DNA can act as a sensor and adaptor to cancer-associated stress. Copy number may be a biomarker with predictive potential.

## Results

### 45S copy number is similar across different mouse tissues and inbred mouse strains, but differs between outbred mice

In the mouse genome, the 45S repeats are located on 5 different chromosomes (12, 15, 16, 18, 19) whereas all the 5S repeats are located on chromosome 8. The copy number for the two repeats co-varies and has been previously estimated using a computational approach from sequence data derived from a collection of laboratory and wild mice, showing a 10-fold range with values for 45S of 31–289 and for 5S of 32–224 [4]. We wanted to extend these findings by developing a ddPCR method to directly measure the copy number of the 45S repeat with high accuracy in commonly used laboratory strains including the inbred strains C57BL/6 and DBA/2J, and the outbred strain CD-1. In addition, we wanted to know whether copy number varied by tissue given different metabolic requirements. Mitochondrial DNA (mtDNA) copy number has been reported to have an inverse relationship with rDNA copy number in human blood samples [17] and is thought to fluctuate with metabolic demand.

**Fig 1:**
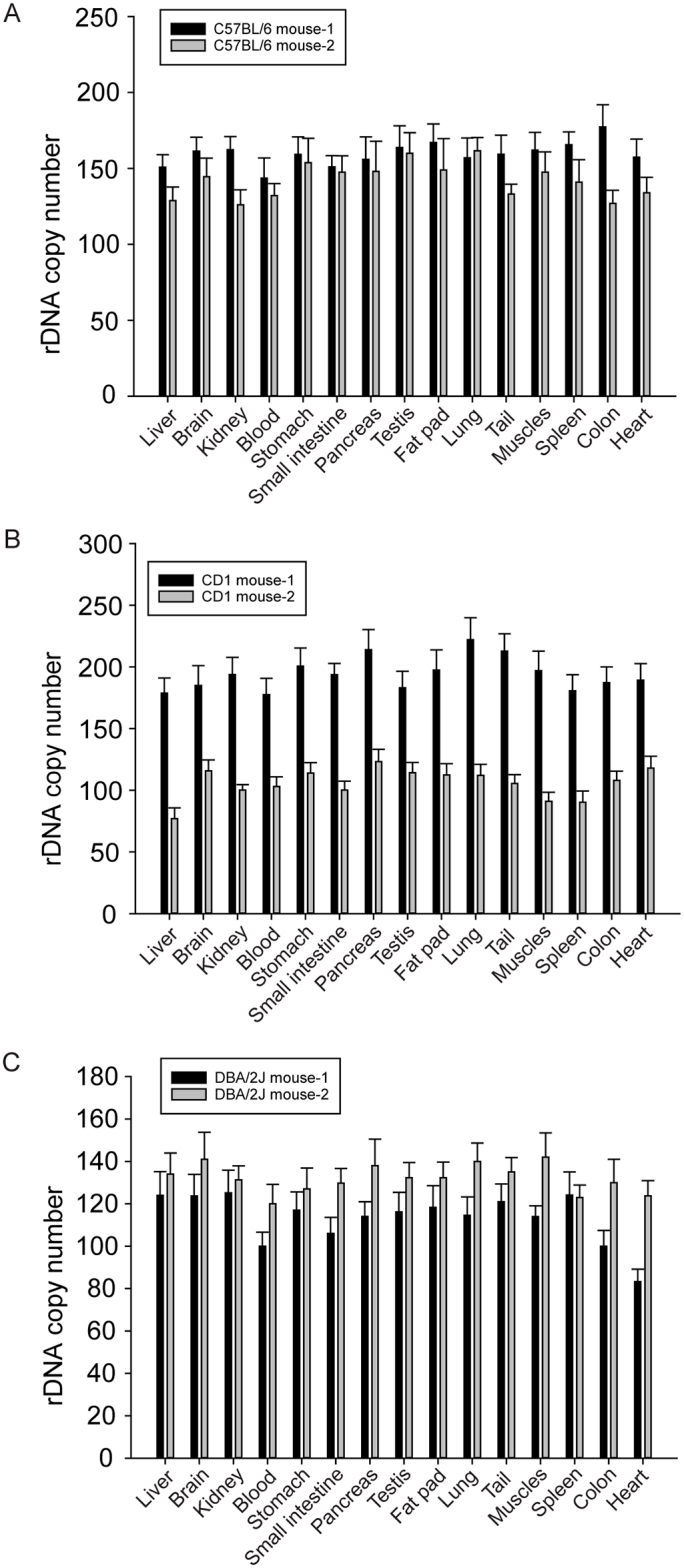
The profiles of rDNA copy number in various tissues from three mouse strains. (A). The pattern of rDNA copy number from 15 different tissues from C57BL/6 mice, (B). The pattern of rDNA copy number from 15 different tissues from CD1 mice, (C). The pattern of rDNA copy number from 15 different tissues from DBA/2J mice.

To answer whether rDNA copy number is variable across different mouse strains, tissues, and organs, we collected genomic DNA (gDNA) from fifteen tissues of three mouse strains, and performed ddPCR. The assay is designed such that the copy number of 45S and the single copy gene *Gapdh1* are measured in the same reaction using fluorescent probes. We found the average copy number for C57BL/6 was 156 based on 57 tissue measurements from eight mice (standard deviation is 13.1), and DBA/2J was 123 based on two mice (standard deviation is 12.3) (Fig 1, S1 Fig). The mouse to mouse variation in the inbred strains was ~10%, with no obvious gender difference (S1 Fig). While the mean copy number for CD-1 was similar (145 based on 5 mice), the variation between individuals was much higher (standard deviation is 43.2), as much as 2-fold in the individuals tested. This demonstrates that mice from inbred strains tend to have a much more similar copy number to each other than individuals from the outbred CD-1 strain. The copy number variation in CD-1 is more like what has been reported in the human population.

Next we addressed how copy number varies across tissues and organs. To first address the level of variation in the ddPCR protocol, we isolated gDNA from the same tissue after dividing it into three samples. In this case the variability is usually less than 5% (S2 Fig). The results suggest that the accuracy of the measurements is appropriate to assess levels of variation higher than 5%. rDNA copy number differs by up to 15% between tissues from the same individual, and this is true in all three strains (Fig 1, S1 Fig). This variation is similar to individual to individual differences from the two inbred strains. From these studies, we conclude that the changes reported in mtDNA copy number in various tissues are not mirrored by equivalent changes in rDNA copy number, since the tissue to tissue variation in a single individual for rDNA is low but for mtDNA is high. Given the result that tissues from the same individual have very similar rDNA copy numbers, this opens the possibility of using matched cancer-normal genome data to explore rDNA copy number using previously developed computational methods that use genome coverage for normalization [17].

### Some cancer genomes have fewer rDNA copies than the matched normal genome

We analyzed high-coverage tumor-normal matched WGS data from 162 individuals across eight tumor types, including 19 childhood acute lymphoblastic leukemia samples (ALL, phs000341) [24], 36 medulloblastoma samples (phs000409) [25], 16 core-binding factor acute myeloid leukemia samples (CBF-AML, phs000414), 40 prostatic neoplasm samples (phs000447) [26], 12 AIDS-related lymphoma samples (phs000530), 12 intestinal neoplasm samples (Liver/Small bowel, phs000579) [27], 13 osteosarcoma samples (phs000699) [28], and 14 esophageal adenocarcinoma samples (phs000598) [29]. The normal WGS data was derived from either blood, solid normal tissue, or germline tissue (S1 Table). We also analyzed the rDNA copy number in WGS data derived from blood of 143 normal individuals (phs000424, GTEx). These datasets were chosen for three reasons: 1) we wanted representation of both solid and blood tumors, 2) WGS data was available and 3) permission to use the data for this analysis was granted. The use of ~16,000 exons for normalization and the comparison of many tumor-normal pairs helps to offset concerns regarding how aneuploidy in the cancer genomes might skew results (see Materials and methods).

**Fig 2:**
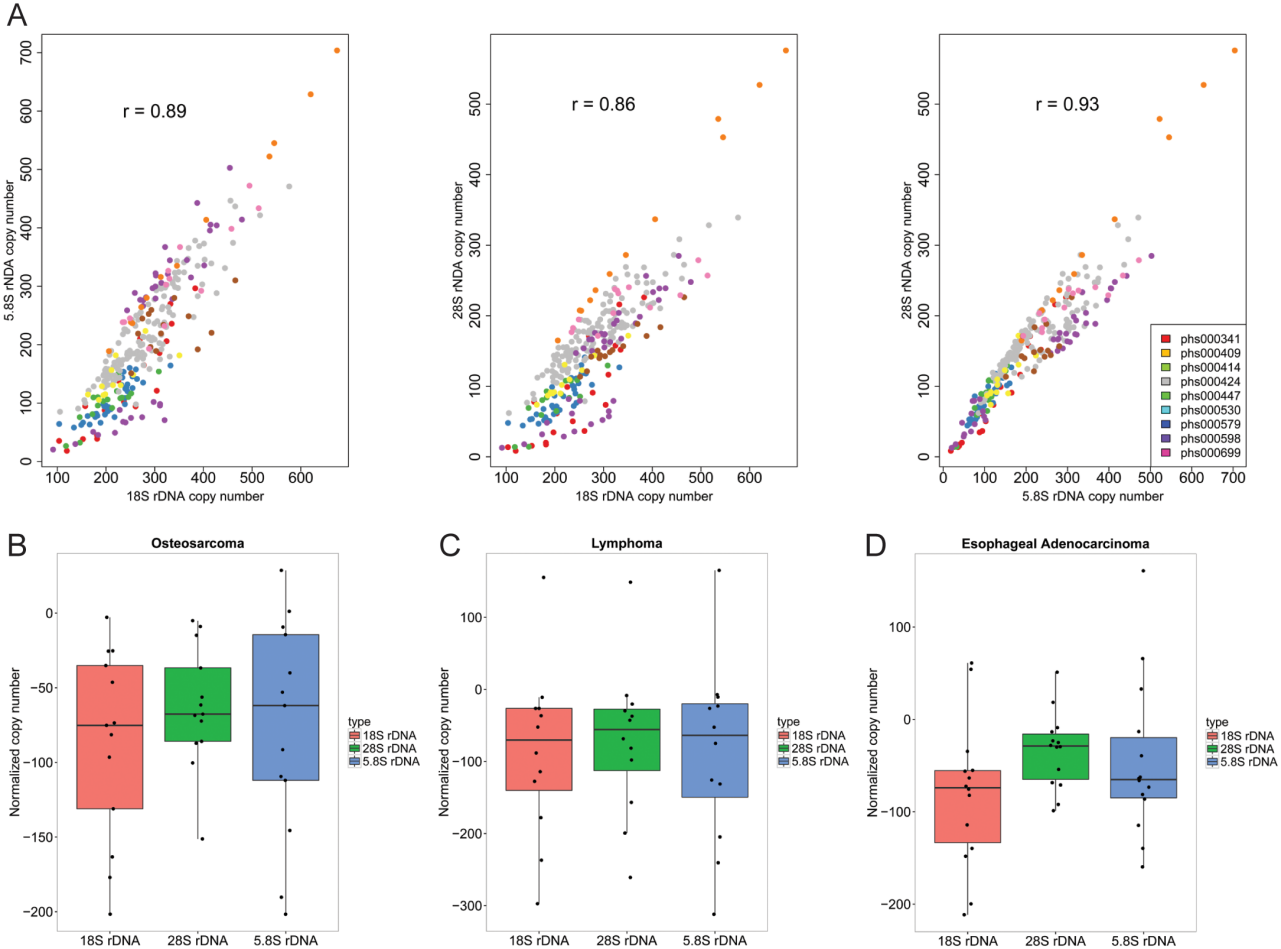
Three cancer genome projects display a lower copy number of 18S, 28S, and 5.8S coding-regions in the 45S rDNA repeats. (A). The pairwise correlations between 18S, 28S, and 5.8S coding sequences are shown for the calculated copy number for all normal samples from the eight cancer projects and GTEx data sets. Both the correlations and the range in copy number is similar to previously published values [17]. (B-D). Normalized copy number for 18S, 28S, and 5.8S rDNA regions is shown for cancer genomes from osteosarcoma, AIDS-related lymphoma, and esophageal adenocarcinoma. (B) 18S_rDNA, P-value = 0.000335; 5.8S_rDNA, P-value = 0.002485; 28S_rDNA, P-value = 0.0001197. (C) 18S_rDNA, P-value = 0.02726; 5.8S_rDNA, P-value = 0.03649; 28S_rDNA, P-value = 0.03703. (D) 18S_rDNA, P-value = 0.00214; 5.8S_rDNA, P-value = 0.0647; 28S_rDNA, P-value = 0.008664.

To first verify the bioinformatics method, we compared our copy number measurements between the three different regions of the 45S gene for all the normal samples. We found good pairwise correlations between 18S, 5.8S, and 28S genes (Fig 2A), similar to those previously reported with this method [17]. We next calculated the copy number for the normal and tumor matched samples. The copy number of 18S, 5.8S, and 28S for the normal samples was subtracted from the tumor for each individual and plotted as the normalized copy number. We found three cancer genome projects, osteosarcoma, AIDS-related lymphoma, and esophageal adenocarcinoma, for which there was a statistically significant reduction in copy number (~70–90 copies) for the tumor genomes (Fig 2B–2D). The other five genome projects did not show significant changes (S3 Fig), suggesting the method can discern loss vs. no loss. We found this result remarkable because it suggests that over the course of tumor development a lower copy number at the rDNA is selected in some cancers. This is opposite to the idea that more copies might be needed in cancer to reach high rates of ribosome biogenesis required for high rates of protein synthesis and proliferation. Instead, some other aspect of high proliferation may select for a lower copy number, for instance, efficient DNA replication. These findings represent the first demonstration that rDNA copies can be lost as part of cancer development.

We used the ~16,000 exons selected for normalization, which are scattered across all chromosomes, to examine whether the chromosomes with rDNA (13, 14, 15, 21, 22) are present at lower levels relative to other chromosomes in the tumor genomes, since this would indicate chromosome loss as one mechanism to lose rDNA copies. These exons were preselected based on the criteria that they were derived from the largest exon (300 bps or larger) of a single gene, mapped uniquely in the genome, and did not have sequence similarity to each other. Using sequencing coverage, we found evidence that chromosomes in the tumor genomes show more variable coverage than in the normal genomes (S4A Fig), presumably reflecting increased aneuploidy. However, the chromosomes with rDNA are not lost at higher rates in genome projects that are positive for rDNA loss compared to the genome projects negative for rDNA loss (S4B Fig). These results suggest preferential loss of rDNA chromosomes is not the predominant mechanism underlying loss of rDNA copies in the cancer genome projects showing a net loss of rDNA copies.

### Cancer genomes with a decrease in rDNA copy number have co-occurring copy number increases in other genes

**Fig 3:**
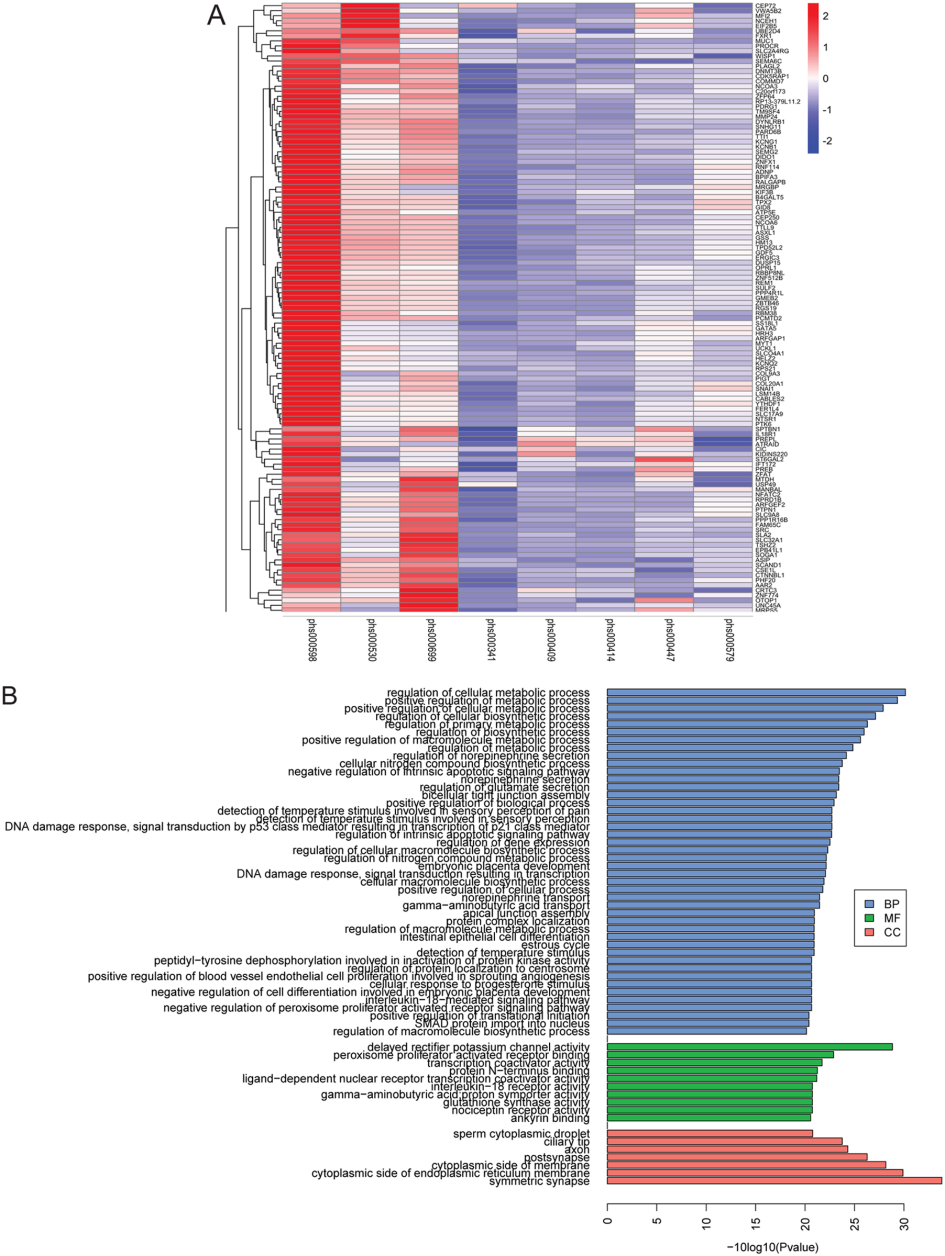
The three low-copy number cancer genome projects have concerted copy number changes in additional genes. (A). The heatmap depicts a cluster of genes with a copy number increase in the three genome projects that have a decrease in rDNA copies. (B). The GO term analysis indicated enrichment for biological processes (BP) such as metabolic process and DNA damage response, molecular functions (MF) such as transcription, and cellular compartments (CC). All terms have a p value less than 0.01 using a hypergeometric test.

To further analyze copy number variation in the cancer genome projects, we used the copy number of the preselected exons used for normalization to discover additional genes whose copy number co-varied with the ribosomal DNA. Exons were selected that displayed a significant copy number difference based on results of a paired t test for all the tumor versus all the normal values for each exon for each genome project (FDR< = 0.05). For the three genome projects with low rDNA copy number in tumor versus normal (phs000530, phs000598, phs000699) there were 353 such exons. We then used hierarchical clustering to identify exons with similar trends in copy number (gain or loss) in the three “positive” projects compared to the five “negative” projects. The “gain” cluster was the most striking, with ~100 exons (Fig 3A). These exons are spread across all chromosomes and are not confined to the chromosomes with 45S genes, indicating global rather than local structural variations accompany the loss of copies. This result suggests there may be a global concerted genomic signature associated with decreased rDNA copies in cancer. The GO terms associated with the genes from which these exons were derived include DNA damage response and metabolic regulation, among others (Fig 3B, S2–S4 Tables), suggesting the increase in copy number of these genes could affect cellular physiology. While none of these terms reach statistical significance when adjusted for multiple hypothesis testing, there are conflicting views on whether this adjustment is necessary for GO term analysis. Unfortunately, RNA-seq data was not available for these cancer genome projects, which would have allowed us to evaluate if there was a corresponding gene expression signature.

### Single nucleotide variation in the 45S repeat

**Fig 4:**
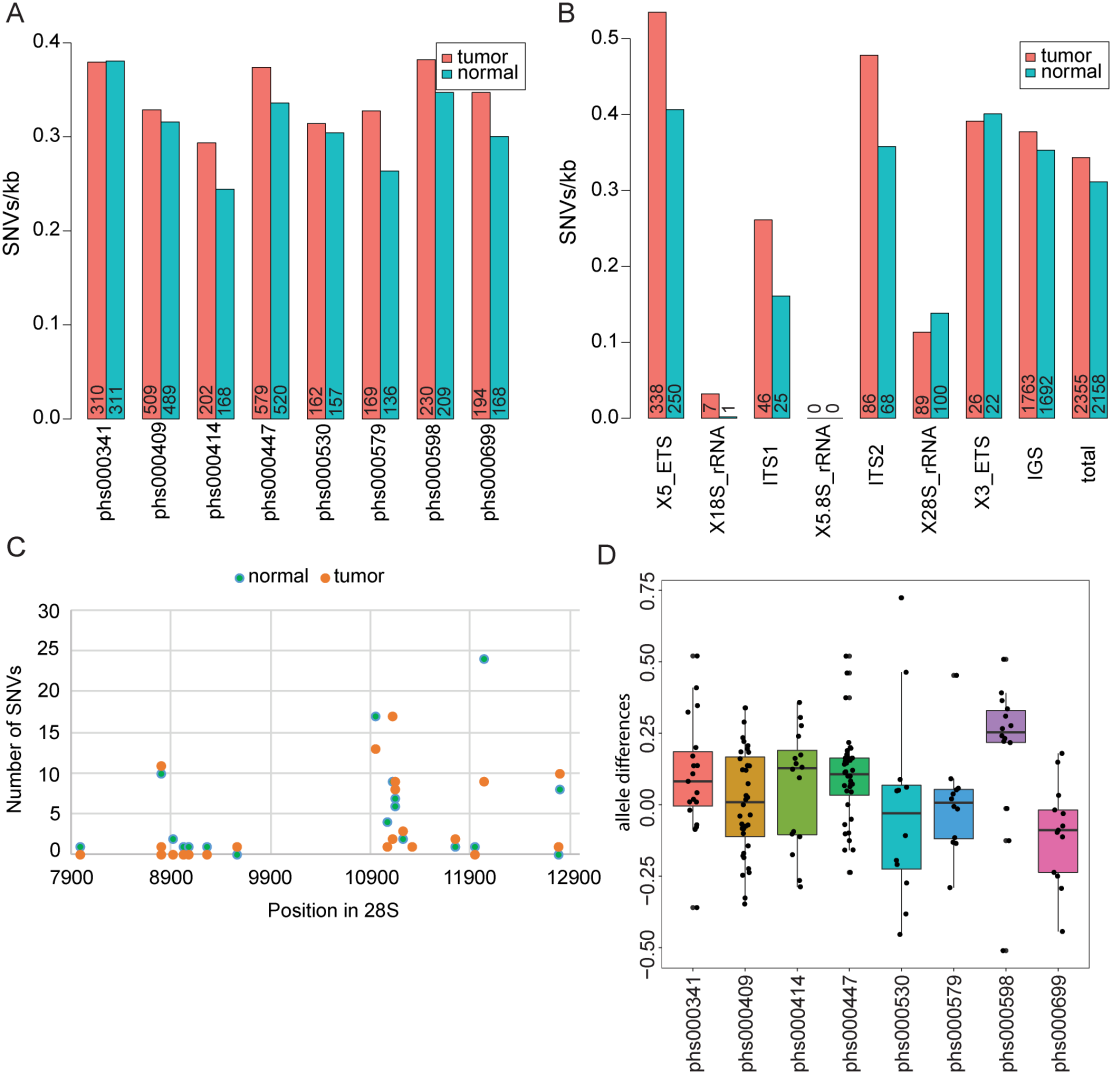
SNV analysis of rDNA loci in the eight cancer genomes from tumor/normal pairs. The match between the test genome and the reference genome sequence was scored at each bp for all genomes. SNVs common to matched pairs were considered “shared” and not included in the analysis in A-C. (A). The plot depicts unique SNVs per kb in the repeat in normal and cancer genomes, with the project number indicated on the x axis, and the allele number on each bar. (B). All unique SNVs for all projects were summed together to depict the SNVs per kb for the different regions of the repeat. The total number of SNVs in each region is annotated on each bar. (C). The number of unique SNVs identified in the 28S region are plotted by position relative to the 45S repeat in GenBank. Hotspots of variation are apparent. (D). The number of alleles for each shared SNVs in each genome pair was used to calculate an average allele number per genome. The average for the normal genome was subtracted from the average calculated for the matched tumor genome to yield the allele difference, plotted by project with results of a t test indicated for each (phs000341, 0.0467, phs000409, 0.7223, phs000414, 0.3458, phs000447, 0.0011, phs000530, 0.8773, phs000579, 0.9782, phs000598, 0.0144, phs000699, 0.0641).

The rDNA represents a prime example of concerted evolution, which refers to the fact that within a species there is very little sequence variation, but there is significant variation between species [7]. The homogeneity is thought to be maintained via frequent and continuous sweeps of recombination. However, variation in the ribosomal DNA sequence has not been assessed in the human genome. We identified single nucleotide variants (SNVs) and indels in tumor and normal genomes relative to a common reference. Indels were rare so we focused on SNVs. Within a single individual, the majority of SNVs (~90%) detected relative to the reference were shared between the tumor and normal genome. However, subtraction of the shared SNVs still yielded several unique SNVs. The number of unique SNVs were similar in tumor and normal genomes across the 8 different projects (Fig 4A, S5 Table). When taken in aggregate, the number of SNVs in the cancer genomes is not higher than in the normal genomes. These results suggest that as cells divide, SNVs can arise at the rDNA and propagate at a similar level in tumor and normal cells.

One might predict the coding portion of the locus to experience selective pressure to generate functional ribosomes for high proliferation. SNVs were binned for each region of the repeat (Fig 4B). Previous work in yeast has suggested the most variable region is the non-transcribed portion, because mutations here might have the least functional consequences [30]. The human repeat is ~30% transcribed (ETS, ITS, 18S, 5.8S, 28S) and ~70% non-transcribed (IGS). We found that the regions encoding 18S and 5.8S had virtually no SNVs in either the tumor or normal genomes, suggesting these sequences cannot tolerate mutations, consistent with a previous report in yeasts [30]. Surprisingly, SNVs were detected in the 28S region, with a similar number in tumor and normal genomes. We detected hotspots for variation; the 189 SNVs were located at only 22 unique positions (Fig 4C). SNVs in the gene encoding 28S rRNA have the potential to affect ribosome function if the corresponding repeats are transcribed.

All non-coding regions of the human repeat had SNVs, including the 5’ and 3’ external transcribed spacer regions, the internal transcribed spacers, as well as the large intergenic region, suggesting these regions can tolerate SNVs. Interestingly, the IGS did not appear to be mutated at a significantly higher rate than the overall repeat (Fig 4B, S6 Table). SNVs in the transcribed non-coding regions have the potential to affect transcription and processing of rRNA but are difficult to functionally evaluate given our limited understanding of the sequence features of the human repeat. In summary, variation in the non-coding region is higher than in the coding region, regardless of transcription. Moreover, the amount of variation is generally similar in the normal and tumor genomes.

Another way to quantify variation that pertains specifically to a multi-copy sequence in the genome is allele number. By this we mean that individual nucleotide positions could have sequence evidence for more than one base, which we refer to as alleles. For the shared SNVs, we evaluated the number of alleles present in the cancer and normal genomes, to ask whether there was differential presence of alleles. We found three genome projects with evidence for more minor alleles in the tumor as compared to normal genomes (Fig 4D). The modest increase observed in prostate neoplasms (phs000447) could be due to the handful of cancer genomes in this project with very high copy number. Most notably, the esophageal adenocarcinoma (EAC) genomes have an increased allele frequency relative to the matched normal genomes (phs000598), despite having a lower copy number. Therefore, in the EAC genomes, there are more alleles of the 45S gene sequence despite fewer copies. The initial publication describing the analysis of the EAC genomes reported mutational complexity with accumulated mutations including activating mutations in *PIK3CA*, and loss of function mutations in *PTEN*, *PIK3R1*, *AKT2*, and *AKT3*, together implicating the mTOR-PTEN pathway in some instances of this cancer [29]. Our results reveal that the mutational complexity of this cancer also encompasses variation in 45S. Overall, our sequence analysis of the human 45S repeats indicates a surprising level of SNVs with newly identified hotspots in the human 28S gene, and some cancer genomes with evidence for increased alleles.

### The Pten^-/-^ mouse leukemia model shows a decrease in rDNA copies in HSC genomes

In the osteosarcoma genome project the tumors are driven by increased mTOR pathway activity, which can occur via multiple different mutations, including loss of *PTEN*, a negative regulator of mTOR [28]. Loss of *PTEN* in prostate cancer is associated with tumor aggression and poor outcome. In fact, mutations that activate the PI3K (phosphatidylinositol-3-kinase)-mTOR pathway are frequently found in many cancers, including AIDS-related lymphomas [31], and esophageal cancer, as mentioned above [29,32,33,34]. *PTEN* is widely known as a tumor suppressor and a guardian of the genome [35]. Because the samples used for the cancer genome projects have a mixed population of mutations, may have genetic instability, and may be derived from mixtures of cell types, we wanted to specifically address whether high mTOR function could influence rDNA copy number in the context of a uniform genetic alteration (loss of *Pten*) in a pure cell population.

We used a mouse model for *Pten*^-/-^ leukemia in the C57Bl6 background to assess rDNA copies in hematopoietic stem cells (HSCs)[36]. *Pten* is excised in bone marrow HSCs postnatally, but remains intact in all tissues other than the progeny of null HSCs. HSCs were isolated from mice using flow sorting of Lineage-, Sca-1+, c-Kit+, and CD34- cells from bone marrow prior to progression to leukemia; these cells are cancer stem cells which will eventually develop to myeloid proliferative disorder and leukemia *in vivo* or upon transplant [37]. These cells are not aneuploid, based on analysis of a model with similar progression to leukemia [38]. *Pten* null cells isolated from bone marrow show more colony forming units and reduced quiescence, demonstrating increased proliferation [36,37]. We verified that loss of *Pten* in HSCs results in a pathway signature indicative of mTOR activation, including higher levels of phosphorylated mTOR, Rps6, and S6K1 (S5 Fig).

**Fig 5:**
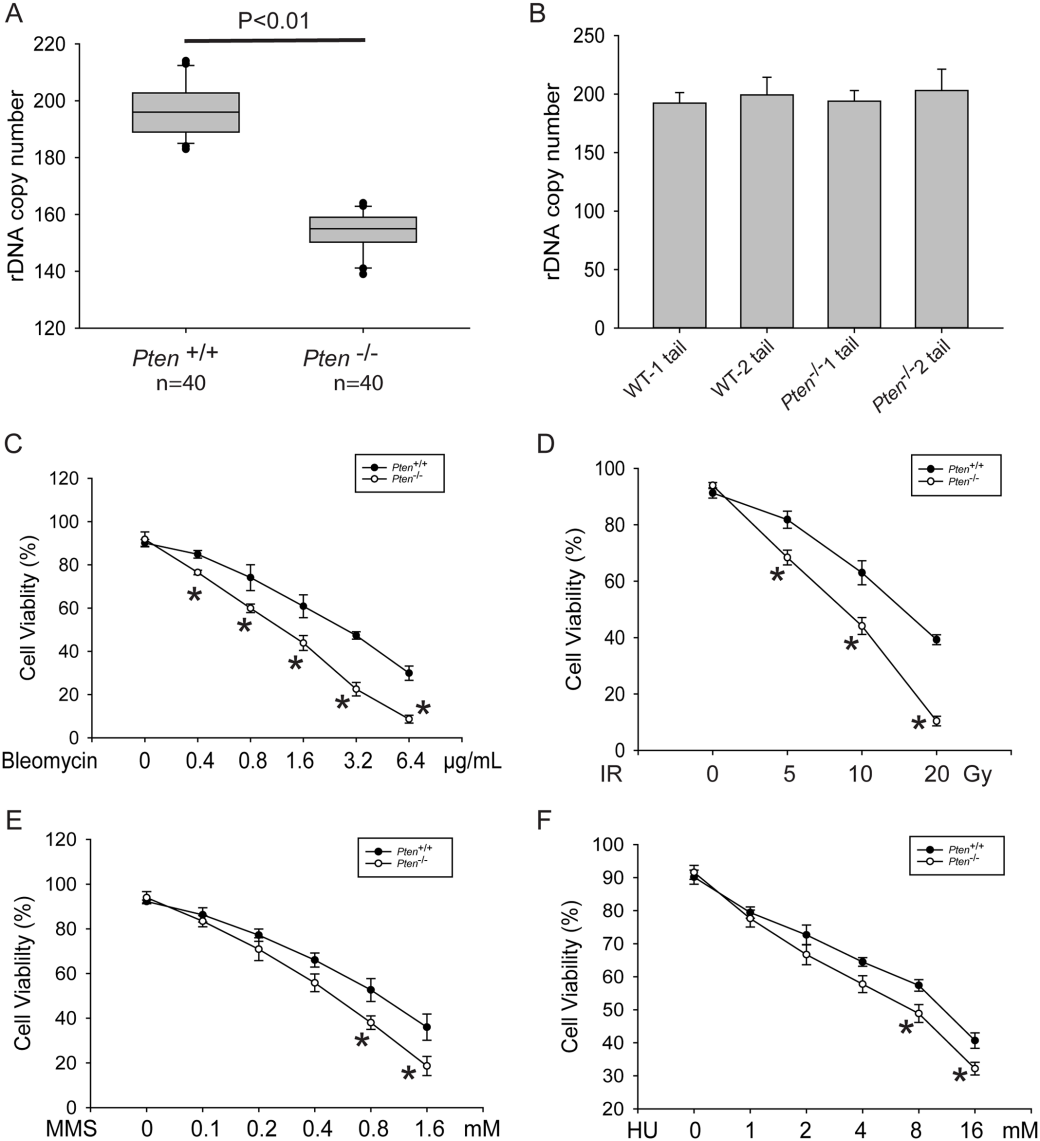
*Pten*^-/-^ HSCs show rDNA copy number reduction and sensitivity to DNA damage. (A). *Pten*^-/-^ null HSCs exhibited a decrease in rDNA copy number compared to matched WT HSCs, samples were derived from two female mice of each genotype with 20 clones each. (B). The rDNA copy number in the mouse tail gDNA samples was similar between individuals, and was also similar to rDNA copy number in WT HSCs. (C-F). HSCs were treated as indicated with bleomycin, ionizing radiation (IR), methyl methanesulfonate (MMS), or hydroxyurea (HU) and the viability was calculated at day five based on total cell number and trypan blue staining. Each dosage was performed in triplicate and the error bars represent standard deviation. Data shown was derived from HSCs from two mice of each genotype. Asterisks represent values for which a t test indicates statistical significance below 0.05.

Remarkably, the *Pten*^-/-^ HSCs have significantly lower copy number compared to two different controls, tail samples from the same mice which retain *Pten* and matched *Pten*^+/+^ HSCs (Fig 5A and 5B). To examine rDNA copy number, we collected WT and *Pten*^-/-^ HSCs from 20 single-cell derived clones from two different mice per genotype (see Materials and methods), and extracted gDNA from these samples for the ddPCR assay. Each *Pten*^-/-^ clone had lost about 40 copies, approximately 20% of their total. HSC clones derived from three additional WT-null pairs showed similar results (S5 Fig), for a total of 5 pairs of female mice. Therefore, loss of *Pten* in HSCs was associated with significant loss of rDNA copies while the copy number in the tails was comparable.

We constructed and sequenced libraries from genomic DNA derived from 20 WT and 20 *Pten*^-/-^ clones derived from two age and sex matched females. Using our computational method, counting of the 5.8S and 18S sequences showed a loss of ~30 copies in the *Pten*^-/-^ clones, and 28S showed a loss of ~20 copies (S6 Fig). Given that the libraries were made using a transposase method that has some bias, the agreement between the computational and ddPCR methods is quite good, and lends confidence in the overall trend toward loss in *Pten*^*-/-*^ HSCs. This trend is consistent with the lower rDNA copies observed in the genomes of osteosarcoma, AIDS-related lymphoma, and esophageal adenocarcinoma. WT and *Pten*^*-/-*^ clones have similar ploidy (S6 Fig), indicating that chromosome loss is not the mechanism by which rDNA is lost in this context. Further analysis of the DNA sequences from the 40 clones revealed 110 SNVs relative to the consensus sequence that were shared between WT and *Pten*^*-/-*^ clones. There were a handful of SNVs that occurred uniquely in *Pten*^*-/-*^ (9) or WT clones (28), with most occurring in the large IGS region (5 for WT and 17 for *Pten*^*-/-*^). No SNVs were detected in 18S or 5.8S, but 3 were detected in 28S. Overall the SNV pattern is similar to that observed in the human genome. Together these results suggest that in some cancers, and specifically in loss of PTEN, loss of rDNA copies may provide some selective advantage for the cancer genome. Furthermore, in the *Pten*^-/-^ HSC model, loss of copies is a relatively early event, prior to progression to leukemia or aneuploidy.

*PTEN* plays a critical role in the maintenance of chromosome stability, preventing double-strand breaks (DSBs) [39]. Deletion of *PTEN* in prostate cancer cells is associated with sensitivity to DNA damaging agents including ionizing radiation, mitomycin-C, UV, H_2_O_2_, and methyl methanesulfonate (MMS)[40]. Loss of *PTEN* in HCT116 colon cancer cell lines confers sensitivity to ionizing radiation [41]. We found that the *Pten*^-/-^ HSCs were more sensitive than WT HSCs to DNA damage, including bleomycin and ionizing radiation (Fig 5C and 5D, S5 Fig), and MMS and hydroxyurea at the higher concentrations tested (Fig 5E and 5F, S5 Fig), consistent with *Pten* protecting against DNA damage in HSCs. HSCs were stained with antibodies to the nucleolar proteins fibrillarin and nucleolin, and nucleoli were imaged. Despite loss of copies, nucleolar size is not significantly different between *Pten*^-/-^ and *Pten*^+/+^ HSCs (S7 and S8 Figs).

**Fig 6:**
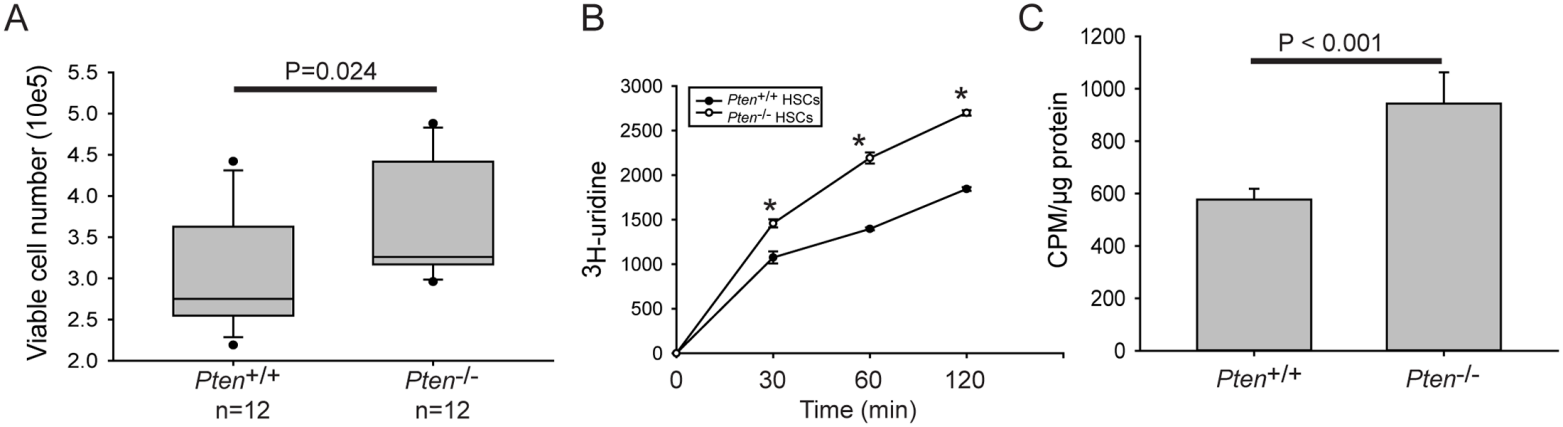
Proliferation, rRNA production, and protein synthesis are robust in *Pten*^-/-^ HSCs. (A). To measure proliferation, HSCs were plated in 100 μl fresh medium, and cultured for 5 days. The viable cell number was measured based on total cell number and trypan blue staining. Data shown was derived from 12 clones derived from two age matched female mice of each genotype. (B). HSCs were pulse labeled with ^3^H-uridine for the time indicated. RNA was isolated with TriZol reagent. 1 μg of total RNA was counted in a Beckman LS 6500 multipurpose scintillation counter to determine new rRNA production. Three clones were labeled to derive the standard deviation from two pairs of mice of each genotype from the same litter. Significance was calculated using an unpaired t test, the asterisk indicates p<0.05. (C). To measure global protein synthesis, HSCs were pre-cultured in medium lacking methionine, and pre-treated with 10 μM MG-132, a proteasome inhibitor, for 1 hour. HSCs were incubated with 30 μCi of ^35^S-methionine for 1 hour. Incorporation of ^35^S-methionine into proteins was quantified in a liquid scintillation counter. Clones were derived from two mice of each genotype, with three replicates per genotype. A t test was performed for statistical significance.

To further examine how loss of copies affects cell function, we compared *Pten*^-/-^ and control HSCs in culture. *Pten*^-/-^ single cell derived colonies have more cells than controls (Fig 6A), consistent with previous reports that *Pten*^-/-^ HSCs proliferate faster. Furthermore, we examined the production of rRNA using ^3^H-uridine incorporation. Because rRNA is the most abundant RNA product, this method has been widely used as a proxy to measure rRNA synthesis. We found that rRNA production is more robust in the *Pten*^-/-^ HSCs compared to *Pten*^+/+^ HSCs despite reduced rDNA copy number (Fig 6B). This result is consistent with previous reports that PTEN normally represses both RNA polymerase I- and RNA polymerase III-dependent transcription [42,43]. Moreover, global protein synthesis, as measured by ^35^S-methionine incorporation, was significantly higher in the *Pten*^-/-^ HSCs (Fig 6C), consistent with a previous study [44]. Therefore, loss of copies and DNA damage sensitivity occurs in the context of more robust proliferation, rRNA production, and protein synthesis.

**Fig 7:**
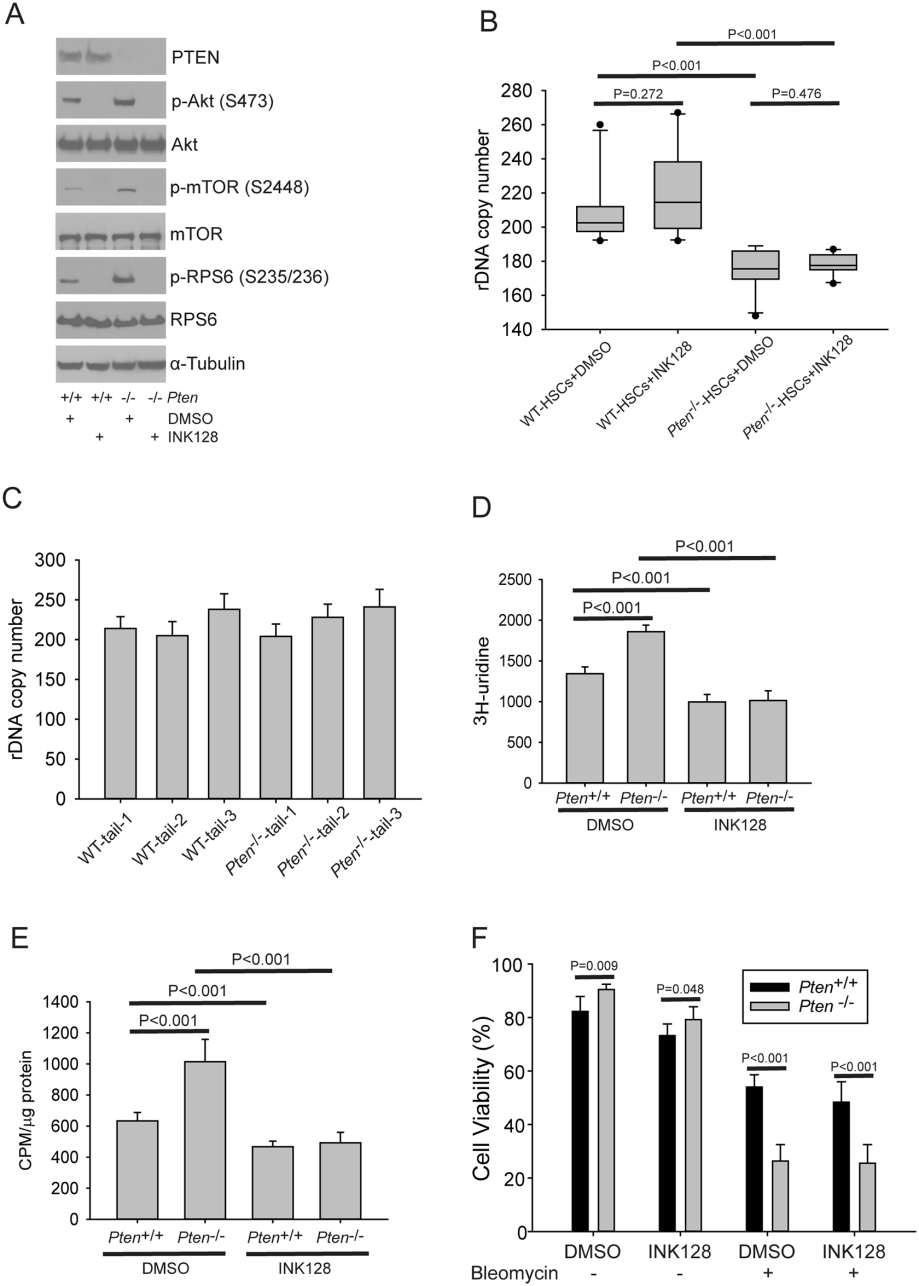
*Pten*^-/-^ HSCs are sensitive to DNA damage when mTOR activity is blocked with INK128. A. Western blot confirming that INK128 treatment (300 nM) blocks mTOR activity in HSCs after 5 days. All HSCs are derived from three age matched males of each genotype. B. *Pten*^-/-^ HSCs exhibited a decrease in rDNA copy number compared to matched WT HSCs, samples were derived from three males of each genotype with 10 clones total, 3 from 2 mice and 4 from the third. (C). The rDNA copy number in the mouse tail gDNA samples was similar between individuals, and was also similar to rDNA copy number in WT HSCs. (D) for D-F, Six independent HSC clones were used per condition, two from each mouse. ^3^H-uridine incorporation was measured after 2 hours of labelling as described in Fig 6B. E. Protein synthesis was measured as described in Fig 6C. F. HSCs were pretreated with INK128 for 2 hrs prior to the addition of bleomycin. INK128 or DMSO and bleomycin were maintained in the culture for the duration of the experiment. Viability was calculated at day five based on total cell number and trypan blue staining.

To determine whether the DNA damage sensitivity was due to fewer copies or the activation of mTOR, we treated HSCs with INK128 to block TOR activity and asked whether the *Pten*^-/-^ HSCs were still sensitive to DNA damage. For these experiments HSCs were derived from 3 age matched pairs of male mice. First we identified a concentration of INK128 that would effectively block TOR activity in the HSCs (Fig 7A). Second, we measured copy number by ddPCR, finding loss of ~30 copies in *Pten*^-/-^ HSCs that occurred independent of treatment with INK128 (Fig 7B). The copy number in the tails of mice was similar across animals and similar to the copy number in the WT HSCs (Fig 7C). Next we measured rRNA production (Fig 7D) and protein synthesis (Fig 7E). These outputs depended on mTOR activity, as expected, and were effectively normalized between the genotypes in the presence of INK128. Together with the results in Fig 6, these results demonstrate similar behavior between HSCs derived from male and female mice, with 8 pairs examined in total. Finally, we examined the sensitivity of the mTOR-blocked HSCs to bleomycin. Importantly, the *Pten*^-/-^ HSCs are more sensitive to bleomycin than the WT HSCs (Fig 7F), suggesting that the sensitivity is not due to differential activation of mTOR, but could derive from the lower copy number.

Finally, we asked whether copy number can change rapidly in human retinal pigment epithelial (RPE) cells grown in culture. RPE cells have a normal karyotype such that the droplet digital PCR method, which uses a single copy reference gene to calculate copy number, can be applied. RPE cells were transfected with 3 different siRNAs to knockdown *PTEN*, or a control siRNA. The knockdown of PTEN was confirmed by Western blot (S9 Fig). The copy number of the RPE cells was determined at 80 hours post-transfection, or after about 3 doublings. Under these conditions the copy number is not altered (S9 Fig). This result suggests that loss may require more doublings, or may require other environmental factors provided in an animal, such as cell-cell competition. This finding suggests that the loss of copies is not an immediate event upon loss of *PTEN* and is consistent with the result in the HSCs that short term treatment with an mTOR inhibitor is not sufficient to alter the copy number.

Our findings suggest that in hematopoietic cancer stem cells, cell growth and ribosome biogenesis can occur robustly with ~30–40 fewer rDNA repeats. Interestingly, in budding yeast, loss of rDNA copies is associated with sensitivity to DNA damage [21]. Our results show that mouse HSCs without *Pten* function have fewer copies and are similarly sensitive to DNA damage. With only half of the rDNA repeats normally transcribed, loss of repeats can be compensated by increasing the fraction transcribed in yeast. However, the binding of factors that normally associate with the inactive repeats to maintain the stability of the locus may be compromised, increasing the sensitivity of yeast with fewer repeats to DNA damage [45]. It remains to be determined whether compensation occurs in the mammalian genome, and whether the absence of silenced repeats could cause DNA damage sensitivity. Nevertheless, copy number may be a useful predictor of DNA damage sensitivity.

## Discussion

We have used both computational approaches and ddPCR to analyze the copy number and sequence of the rDNA in mammalian cells. We find tissue to tissue variation in copy number in a single mouse is relatively low, as is individual to individual variation within an inbred strain. However, in an outbred mouse strain, the level of variation is higher, more resembling the situation in human genomes. We report for the first time that some cancer genomes, and in particular genomes associated with high mTOR activity, tend to have fewer copies than the matched normal genomes, a finding replicated in mouse cancer stem cells, and consistent with a previous report demonstrating transgenerational loss of rDNA copies in *D*. *melanogaster* with overactive TOR [13]. The low copy cancer genomes show concerted copy number changes in additional genes, suggesting a structural signature for these genomes. rDNA sequence variation can also occur in cancer genomes. Low copy in the *Pten*^-/-^ HSCs is associated with sensitivity to DNA damage, extending previous reports that *PTEN* guards against genome instability. Our results suggest that copy number and sequence can change in the mammalian genome in cancer, and that loss of copies may have both costs and benefits. Future studies further analyzing the mechanisms and functional consequences of genomic alterations at the rDNA in mammals are warranted. Given our findings, it seems possible that ribosomal DNA could exhibit changes in other contexts.

Loss of rDNA repeats may affect genome function and chromosomal processes via the release of protein factors. The rDNA locus houses many pluripotency factors in mammalian cells, including Oct4 [46]. The rDNA also contains binding sites for Myc [47], a key transcription factor for proliferation that can also affect DNA replication [48], and CTCF [49], a key chromosome organization protein. The rDNA chromatin contains many different histone modifications [49], which could sequester chromatin readers and writers. Finally, nucleolar associated domains are enriched in regions displaying heterochromatin signatures in *Arabidopsis* [50] and rDNA copy number titrates position effect variegation, a heterochromatin based silencing phenomenon in *Drosophila* [23]. Losing repeats has the potential to liberate factors for re-distribution to the rest of the genome which could affect chromosome organization, gene expression, and replication.

Interestingly, PTEN has been shown to directly control the function of the DNA replication factor MCM2 during DNA replication stress [51]. Replication stress occurs at the rDNA and has been reported as a potent driver of functional decline in HSCs [52]. In a mouse model for cancer driven by deficiency in *Mcm2*, genomic deletions can occur [53]. Spontaneous DSBs also occur at higher levels upon loss of PTEN [39]. Together these findings suggest that one possible reason that rDNA repeats are lost in the absence of PTEN is that DSBs and replication stress are handled without MCM2 function, and repair events are required. The repair events that result in deletions may be selected for, since the rDNA is difficult to replicate and loss of repeats might facilitate a successful cell cycle. We suggest that rDNA may both sense and adapt to genomic stress, with PTEN-mTOR normally guarding against copy loss.

Extrachromosomal expansions of rDNA have been reported in frog oocytes [10]. This expansion is thought to facilitate the production of rRNA for ribosome biogenesis and the translational requirements in these cells. Based on this finding we predicted that cancer cells might expand the repeats due to similar requirements. Instead, we found recurrent evidence for contractions. We note that there is a key difference between the oocyte and the cancer cell—rounds of DNA replication. We speculate that the requirement to replicate the rDNA in cancer may select for the loss of repeats while the lack of replication in the oocyte may allow the expansion to be tolerated. The timing of the loss of the repeats relative to the development of cancer cannot be determined from the cancer genome projects. However, in the mouse HSCs loss occurs prior to the development of leukemia and aneuploidy, at the stage of a cancer stem cell. Further characterization of the selective forces and timing of loss will be interesting questions for the future.

Linking the rDNA to both genome stability and ribosome biogenesis may enable it to act as a critical molecular sensor. Changes in translation are associated with cancer [54]. Myc and PI3K-AKT-mTOR are major oncogenic signaling pathways that promote reprogramming of translation. Studies to date have focused on mRNA regulatory elements, tRNA function and codon usage bias, and adaptations to stress that affect translation, but not ribosomal DNA copy number as a factor under the control of TOR signaling. Protein synthesis is quite tightly regulated in HSCs and defects that arise via loss of *Pten* or silencing of rDNA repeats cause functional decline and aging [44,52]. Our study suggests that rDNA copy number and sequence can be altered in human cancers. HSCs with fewer copies are more sensitive to DNA damage, but are not compromised for rRNA production, proliferation, or protein synthesis. We speculate that the extra inactive copies may normally serve in part to counteract rDNA instability. Together, our data and others show that multiple mechanisms regulate protein synthesis and genome stability to control aging and prevent leukemogenesis in HSCs, and that these processes may be linked using the rDNA as a sensor.

In summary, the rDNA copy number and sequence can change in cancer, with high mTOR activity associated with contractions and DNA damage sensitivity. With this recognition comes the possibility to target these loci. Cancer stem cells with low copy number may be more sensitive to DNA damaging agents. We speculate that this single copy number measurement could be used as a proxy detector for the variety of mutations that can occur in the PI3K-AKT-mTOR pathway in cancer and a predictor of whether DNA damaging drugs would selectively target the cancer stem cells. These findings may be applicable to cancer diagnosis and therapeutic choice.

## Materials and methods

### Ethics statement

All animals were handled in strict accordance with good animal practice as defined by the relevant national and/or local animal welfare bodies, and all animal work was approved by the Stowers Institute for Medical Research, Institutional Animal Care and Use Committee.

### Isolation of gDNA samples from various mouse tissues and mouse HSCs

Animals were sacrificed by carbon dioxide administration. All mice were 8 week old males or females, as noted. Fifteen tissues were isolated from three mouse strains including C57BL/6, DBA/2J, CD1. All the tissues were stored at -80°C. Most tissues or organs were cut into 2–3 small pieces to purify gDNA. For mouse HSCs, about 10^5^ cells were used to extract gDNA. gDNA samples were extracted using the Maxwell^®^ 16 Tissue DNA Purification Kit in Promega Corporation. The concentration of gDNA samples was measured by Qubit dsDNA HS Assay. For digital droplet PCR, 1 ng template was used per reaction.

### Analysis of rDNA copy number

Digital droplet PCR was used to measure rDNA copy number, performed per the manufacturer’s protocol (Bio-Rad). Briefly, isolated gDNA was digested with the restriction endonuclease Hae III. Reaction mixtures were made with target copy number variable sequence (45S rDNA) and internal control (*Gapdh1* for mouse, *TBP* for human). Droplet generation was performed, followed by endpoint PCR. Each PCR product is detected by a fluorescent probe. Droplets were read by QX200 droplet reader, and quantitation was performed using Quantasoft software.

### Collection of mouse HSCs

The *Pten* tamoxifen-inducible SCL-Cre mouse model has been previously described [36]. In brief, mice at 6–8 weeks of age were given 2 mg tamoxifen for 5 days. The 8 pairs of mice used for these experiments were age and sex matched. Both WT and *Pten^loxP/loxP^* (Lesche); *HSC-Scl-Cre-Er^T^*^+^ (Göthert) were treated with tamoxifen. HSCs were collected 12 days after the final injection. At this point these cells are considered leukemic stem cells. The timing of development of a clinically defined leukemia *in vivo* varies in this model, generally occurring within 3–4 months post-induction, but sometimes sooner. The *Pten*^-/-^ HSC cause leukemia development after transplant. HSCs were sorted (in this case defined as lineage negative, Sca-1+, c-Kit+, and CD34- cells) by flow cytometry into methylcellulose semi-solid medium (M3434 media from Stem Cell Tech.). Single HSCs are sorted into individual wells in a 96-well plate. These single cells will (in about 35–50% of the wells) form a large colony of mainly myeloid and erythroid hematopoietic cells all derived from the single HSC. The individual colonies are harvested by incubating a flooded well in PBS for 20–30 minutes, and pipetting up and down to disassociate the colony.

### Protein isolation, SDS PAGE and Western blotting analysis

Cells were rinsed once with ice-cold PBS and lysed in ice-cold lysis buffer (buffer A: 50 mM HEPES-KOH (pH 7.4), 2 mM EDTA, 10 mM pyrophosphate, 10 mM β-glycerophosphate, 40 mM NaCl, 1% Trition X-100 and one tablet of EDTA-free protease inhibitors (Roche) per 25 mL). The soluble fraction of the cell lysate was isolated by centrifugation at 12,000 g for 10 min. Sodium dodecyl sulfate-polyacrylamide gel electrophoresis (SDS-PAGE) was performed using NuPAGE Novex 4%–12% Bis-Tris precast gels (Invitrogen). Western blotting was performed per standard protocol using a nitrocellulose (Whatman, Protran) membrane. The following antibodies were used: mTOR (Cell Signaling Technology, #2972), phospho-mTOR (Ser2448) (Cell Signaling Technology, #2971), phospho-S6K1 (Thr389) (Abcam company, ab126818), S6K1 (Cell Signaling Technology, #9202), phospho-RPS6 (Ser235/236) (Cell Signaling Technology, #2211), RPS6 (Santa Cruz Biotechnology, sc-74459), α-tubulin (Sigma Inc., T6199), phospho-Akt (Ser473) (Cell Signaling Technology, #4060), Akt (Cell Signaling Technology, #9272), PTEN (Cell Signaling Technology, #9188). Secondary antibodies were HRP linked, anti-rabbit IgG (from donkey) and anti-mouse IgG (from sheep), (GE Healthcare, NA934V, NA931V, and NA935V, respectively).

### Immunofluorescence staining of mouse HSCs

The 10^5^−10^6^ *Pten*^+/+^ and *Pten*^-/-^ HSCs were seeded on 8-well chamber slides, with poly-L-lysine pre-coating overnight at 4°C. The HSCs were cultured for 24 hours for cell attachment. Next, the samples were washed in PBS, fixed for 10 min at room temperature 20–22°C with 4% paraformaldehyde, permeabilized for 5 min in PBS containing 0.5% Triton X-100 and washed in PBS. After blocking for 30 min in PBS containing 1% BSA at room temperature, the preparations were incubated overnight at 4°C with the following antibodies diluted in PBS containing 1% BSA: mouse anti-fibrillarin with 1:200 dilution, rabbit anti-nucleolin (H-250; Santa Cruz) with 1:200 dilution. The coverslips were then washed in PBS and incubated for 30 min at room temperature with the secondary antibodies Alexa Fluor 488 goat anti-mouse and Alexa Fluor 555 donkey anti-rabbit (Molecular Probes) diluted in PBS containing 1% BSA and then washed in PBS. The coverslips were mounted on slides and analyzed by fluorescence imaging with a Zeiss Axioplan II confocal microscope. The quantification of nucleolar size was performed in the fibrillarin and nucleolin stained regions using Image J software. Nucleolar size distributions and average sizes were determined using custom ImageJ plugins essentially as in [55] but with minor modifications to account for higher image resolution. Firstly, confocal images were maximum intensity projected. Briefly, a rolling ball background with a radius of 5 pixels was subtracted from each image. Next, images were smoothed with a Gaussian blur of 2 pixels (standard deviation). Finally, images were thresholded at 0.25 times the maximum processed intensity in the image. Each resulting distinct spot was considered a nucleolar region for area measurements.

### Metabolic labeling to measure rRNA production in mouse HSCs

Methods for rRNA labeling were derived from a previous report [55,56]. The cultured *Pten*^+/+^ and *Pten*^-/-^ HSCs (10^5^−10^6^) were washed in PBS twice, and switched to fresh Dulbecco’s Modified Eagle’s Medium (Sigma) supplemented with 200 μM L-cysteine (BSA, Sigma), 50 μM 2-mercaptoethanol (Sigma), 1 mM L-glutamine (Gibco) and 0.1% bovine serum albumin (Sigma). HSCs were pulse labeled with ^3^H-uridine (5 μCi) for the indicated amount of time (0, 30, 60, 120 min) per sample. Total RNA was isolated with TriZol reagent (Invitrogen, U.S.A) and the concentration of each RNA sample was measured by Qubit RNA assay. 1 μg of each sample was counted in a Beckman LS 6500 multipurpose scintillation counter to determine new rRNA production based on ^3^H-uridine incorporation. Three replicates were used to derive the standard deviation from two pairs of mice (WT and *Pten*^-/-^) from the same litter. Significance was calculated using an unpaired t test.

### Metabolic labeling to measure protein synthesis in mouse HSCs

*Pten*^+/+^ and *Pten*^-/-^ HSCs (10^5^−10^6^) were plated in 100 μl of methionine/ cysteine -free Dulbecco’s Modified Eagle’s Medium (Sigma) supplemented with 200 μM L-cysteine (Sigma), 50 μM 2-mercaptoethanol (Sigma), 1 mM L-glutamine (Gibco) and 0.1% BSA. The HSCs were pre-cultured with the fresh medium for one hour to deplete endogenous methionine. HSCs were pre-treated with 10 μM MG-132, a proteasome inhibitor, for 1 hour, and then were labeled with 30 μCi of ^35^S-methionine for 1 hour. Cells were lysed in RIPA buffer and proteins were precipitated by the addition of hot 10% TCA. After centrifugation, the precipitate was washed twice in acetone. The precipitate was dissolved in 100 μL of 1% SDS and heated at 95°C for 10 min. An aliquot of the SDS extract was counted in Esoscint for ^35^S radioactivity in a liquid scintillation counter to determine the amount of ^35^S-methionine incorporated into proteins.

### Cell culture and drug treatment

To measure growth in the presence of DNA damage, mouse HSCs (3X10^4^) were plated in 100 μl fresh medium containing various DNA damage stresses at the indicated dosages. After culturing for five days, cell viability was assessed based on total cell number and trypan blue staining.

### Computational analysis of cancer genome projects

Cancer genome data was obtained with permission from dbGaP. The analysis for rDNA copy number is similar to a previously published method [17]. Briefly, the human consensus 45S rDNA sequences was obtained from NCBI (accession: U13369). Raw fastq whole-genome DNA sequence reads were downloaded from dbGaP. Reads were mapped to the 45S locus, and a set of 16022 pre-selected putative single-copy exons (the largest from each gene) using Bowtie2 v2.1.0 with default parameters. Only concordant read pairs are kept in the down-stream analysis. The rDNA copy number of 18S, 5.8S, and 28S was calculated as the mean coverage in the respective regions. To make samples comparable to each other, the rDNA copy number was further normalized to the background genome coverage, which is calculated as the median coverage of the single copy exons.

For sequence analysis, the predominant sequence of each individual genome was compared to the human reference sequence for 45S. Each nucleotide position was either a match to the consensus 45S sequence, or if not a match, then the position was called a SNV. SNVs were called using samtools mpileup (v1.2) after the duplicated reads were removed from the aligned read files. SNVs were further filtered with the quality score (>20, “PASS”). If the SNVs of the cancer and normal matched genome pair were identical, this was termed a shared SNV. If the SNV was not identical, this position was termed a unique SNV. For the shared SNVs in a pair, each position was evaluated for sequence evidence of 1–4 different nucleotides. The allele numbers for the shared SNVs were used to calculate an average allele number for each genome. The average of the number of alleles at the shared SNVs for normal was subtracted from tumor to generate the allele difference for each matched pair, plotted as an individual point in the box plot. GO terms were calculated using the R package GOstats.

### DNA sequencing

DNAseq libraries were generated from 1 ng of genomic DNA as assessed by the Qubit 2.0 Fluorometer (Life Technologies). Libraries were made according to the manufacturer’s directions for the Nextera XT Library Prep Kit (Illumina, Inc.) and purified using the Agencourt AMPure XP system (Beckman Coulter). Resulting libraries were checked for quality and quantity using the LabChip GX (Perkin Elmer) and Qubit. Equal molar libraries were pooled, re-quantified and sequenced as 125 bp paired reads on the Illumina HiSeq 2500 instrument using HiSeq Control Software 2.2.58. Following sequencing, Illumina Primary Analysis version RTA 1.18.64 and Secondary Analysis version bcl2fastq2 v2.17 were run to demultiplex reads for all libraries and generate FASTQ files.

### Transfection of RPE cells

Human RPE1 cells (hTERT-immortalized retinal pigment epithelial cell line from ATCC), at low passage number and ~70% cell confluence, were transfected by three different *PTEN*-siRNAs or the control siRNAs (Ambion) using Lipofectamine RNAiMAX Transfection Reagent from Thermo Fisher Scientific company. 24 hours post-transfection, cells were placed in fresh Dulbecco's Modified Eagle Medium plus 10% Fetal Bovine Serum. At 80 hours post-transfection, cells were harvested. Genomic DNA was extracted using the Maxwell^®^ 16 Blood DNA Purification Kit from Promega Corporation. The DNA concentration was measured using the Qubit dsDNA HS assay, and normalized to 1ng/μL.

## Supporting information

S1 Fig The rDNA copy number analysis of four tissues from three additional mice. (A). The rDNA copy numbers are similar in four tissues from three C57BL/6 mice, (B). The rDNA copy numbers are similar in four tissues from three CD1 mice, but have significant differences between individuals in the outbred strain.
(TIF)

S2 Fig Variability from three independent experiments per single tissue from the same individual was measured for the ddPCR assay. (A). The rDNA copy number is similar in different portions of four tissues from two C57BL/6 mice. (B). The rDNA copy number is similar in different portions of tissues from two DBA/2J mice. (C). The rDNA copy number is similar in different portions of three tissues from two CD1 mice.
(TIF)

S3 Fig Copy number profiles of 18S, 5.8S, 28S rDNA coding-regions in five cancer genome projects that do not exhibit copy number differences compared to matched normal samples. (A) Child Acute lymphoblastic leukemia, (B) Core binding factor acute myeloid leukemia, (C) Intestinal Neoplasms (Liver/Small bowel), (D) Medulloblastoma, (E) Prostatic neoplasms.
(TIF)

S4 Fig rDNA containing chromosomes are not lost at higher rates than normal chromosomes. A. Normalized coverage of each chromosome for tumor (left) and normal (right) genome for each person is shown in a heatmap for each of the 3 projects for which we calculated loss of copies of rDNA. The tumor genomes show more variable coverage than the normal genomes, indicating aneuploidy, as might be expected. B. For the 5 human chromosomes containing rDNA (13, 14, 15, 21, 22), the normalized coverage is plotted for tumor and normal genomes for all genome projects negative for loss of rDNA (rDNA chromosomes-negative) and positive for loss of rDNA (rDNA chromosomes-positive). While there is clearly more variable coverage in the tumor genomes for these chromosomes in both positive and negative genome projects, there is not a trend toward coverage loss in the positive projects that could account for the loss of rDNA.
(TIF)

S5 Fig Confirmation of mTOR activation in *Pten*^-/-^ HSCs and decrease in copy number accompanied by increase in damage sensitivity. (A) Western blotting for various indicators of mTOR activity was performed to confirm that loss of PTEN results in activation of mTOR activity in HSCs. (B) *Pten*^-/-^ HSCs exhibited a decrease in rDNA copy number compared to matched WT HSCs, samples were derived from 3 mice of each genotype with 8–12 clones each (n = 28 for *Pten*^+/+^ HSCs, and n = 33 for *Pten*^-/-^ HSCs). (C). The rDNA copy number in the gDNA derived from tail samples was similar between the WT mice used to derive the HSC clones used in (B), and was also similar to rDNA copy number in *Pten*^+/+^ HSC clones derived from these mice. Tail DNA was not available for the mice used to derive the HSC clones in (B). (D-G). HSCs were treated as indicated and the viability was calculated at day five based on total cell number and trypan blue staining. Each dosage was performed in triplicate and the error bars represent standard deviation. Data shown was derived from HSCs from a single mouse of each genotype. Asterisks represent values for which a t test indicates statistical significance below 0.05.
(TIF)

S6 Fig DNA seq verifies loss of rDNA in *Pten*^-/-^ HSC clones and euploidy. A. DNA was isolated from 20 WT and 20 *Pten*^-/-^ HSC clones. Libraries were made using the Nextera kit and subjected to sequencing at low coverage. We employed our computational pipeline developed for the human cancer genome data to analyze these samples. (A) We found a loss of 20–30 copies of rDNA in the *Pten*^-/-^ clones, depending on the sequence counted (5.8S, 18S, or 28S). For each of the 3 sequences, a t test showed statistical significance lower than 0.0001. (B) ~16,000 single copy exons were used to examine ploidy. Coverage is plotted as a heat map by the average coverage for each chromosome for each HSC clone, with the genotype indicated. There are no obvious differences in chromosome coverage between the WT and *Pten*^-/-^ clones, indicating that the WT and *Pten*^-/-^ clones are similarly euploid.
(TIF)

S7 Fig Immunofluorescence staining of nucleolar proteins fibrillarin and nucleolin, along with DAPI staining of DNA, in WT and *Pten*^-/-^ HSCs. (TIF)

S8 Fig Nucleolar size measurements of the immunofluorescence staining of nucleolin (A) and fibrillarin (B) in WT and *Pten*^-/-^ HSCs. The average density distribution of nucleolar area is plotted. (C) Average area of fibrillarin staining was quantified and compared. About 40 cells were quantified for each sample with seven replicates.
(TIF)

S9 Fig Knockdown of *PTEN* with siRNA in RPE cells does not affect rDNA copy number. A. RPE cells were transfected with three different siRNAs to *PTEN*. The efficiency of knockdown was monitored by Western blot after 80 hours. Other proteins in the mTOR pathway were also monitored by Western blot. B. ddPCR was performed on genomic DNA isolated from cultures 80 hours post-transfection. ddPCR was performed for the human 45S gene with *TBP* as the single copy reference gene. Error bars represent the standard deviation of triplicate reactions.
(TIF)

S1 Table Summary of cancer genome data used in this study. (XLSX)

S2 Table GO term analysis by biological process for the gene list with copy number variation co-occurring with rDNA loss in tumor genomes. For Table S6-8, “count” is the number of genes affected in our data set, “size” is the number of genes assigned to that GO term.
(XLSX)

S3 Table GO term analysis by molecular function for the gene list with copy number variation co-occuring with rDNA loss in tumor genomes. (XLSX)

S4 Table GO term analysis by cell compartment for the gene list with copy number variation co-occuring with rDNA loss in tumor genomes. (XLSX)

S5 Table The total number of unique SNVs for tumor-normal paired samples by position in the repeat for each cancer genome project. (XLSX)

S6 Table The percentage of unique SNVs per kb for tumor-normal paired samples by position in the repeat for each cancer genome project. (XLSX)

## References

[1] McStay B (2016) Nucleolar organizer regions: genomic 'dark matter' requiring illumination. Genes Dev 30: 1598–1610. doi: 10.1101/gad.283838.116 27474438 10.1101/gad.283838.116 PMC4973289

[2] Forsburg SL (2013) The CINs of the centromere. Biochem Soc Trans 41: 1706–1711. doi: 10.1042/BST20130146 24256279 10.1042/BST20130146 PMC3898698

[3] De Lange T (2005) Telomere-related genome instability in cancer. Cold Spring Harb Symp Quant Biol 70: 197–204. doi: 10.1101/sqb.2005.70.032 16869754 10.1101/sqb.2005.70.032

[4] Gibbons JG, Branco AT, Godinho SA, Yu S, Lemos B (2015) Concerted copy number variation balances ribosomal DNA dosage in human and mouse genomes. Proc Natl Acad Sci U S A 112: 2485–2490. doi: 10.1073/pnas.1416878112 25583482 10.1073/pnas.1416878112 PMC4345604

[5] Long EO, Dawid IB (1980) Repeated genes in eukaryotes. Annu Rev Biochem 49: 727–764. doi: 10.1146/annurev.bi.49.070180.003455 6996571 10.1146/annurev.bi.49.070180.003455

[6] Stults DM, Killen MW, Pierce HH, Pierce AJ (2008) Genomic architecture and inheritance of human ribosomal RNA gene clusters. Genome Res 18: 13–18. doi: 10.1101/gr.6858507 18025267 10.1101/gr.6858507 PMC2134781

[7] Eickbush TH, Eickbush DG (2007) Finely orchestrated movements: evolution of the ribosomal RNA genes. Genetics 175: 477–485. doi: 10.1534/genetics.107.071399 17322354 10.1534/genetics.107.071399 PMC1800602

[8] Caburet S, Conti C, Schurra C, Lebofsky R, Edelstein SJ, et al (2005) Human ribosomal RNA gene arrays display a broad range of palindromic structures. Genome Res 15: 1079–1085. doi: 10.1101/gr.3970105 16024823 10.1101/gr.3970105 PMC1182220

[9] Laferte A, Favry E, Sentenac A, Riva M, Carles C, et al (2006) The transcriptional activity of RNA polymerase I is a key determinant for the level of all ribosome components. Genes Dev 20: 2030–2040. doi: 10.1101/gad.386106 16882981 10.1101/gad.386106 PMC1536055

[10] Brown DD, Dawid IB (1968) Specific gene amplification in oocytes. Oocyte nuclei contain extrachromosomal replicas of the genes for ribosomal RNA. Science 160: 272–280. 4867987 10.1126/science.160.3825.272

[11] Gall JG (1974) Free ribosomal RNA genes in the macronucleus of Tetrahymena. Proc Natl Acad Sci U S A 71: 3078–3081. 4528573 10.1073/pnas.71.8.3078 PMC388624

[12] Stults DM, Killen MW, Williamson EP, Hourigan JS, Vargas HD, et al (2009) Human rRNA gene clusters are recombinational hotspots in cancer. Cancer Res 69: 9096–9104. doi: 10.1158/0008-5472.CAN-09-2680 19920195 10.1158/0008-5472.CAN-09-2680

[13] Aldrich JC, Maggert KA (2015) Transgenerational inheritance of diet-induced genome rearrangements in Drosophila. PLoS Genet 11: e1005148 doi: 10.1371/journal.pgen.1005148 25885886 10.1371/journal.pgen.1005148 PMC4401788

[14] Jack CV, Cruz C, Hull RM, Keller MA, Ralser M, et al (2015) Regulation of ribosomal DNA amplification by the TOR pathway. Proc Natl Acad Sci U S A 112: 9674–9679. doi: 10.1073/pnas.1505015112 26195783 10.1073/pnas.1505015112 PMC4534215

[15] Paredes S, Branco AT, Hartl DL, Maggert KA, Lemos B (2011) Ribosomal DNA deletions modulate genome-wide gene expression: "rDNA-sensitive" genes and natural variation. PLoS Genet 7: e1001376 doi: 10.1371/journal.pgen.1001376 21533076 10.1371/journal.pgen.1001376 PMC3080856

[16] Paredes S, Maggert KA (2009) Ribosomal DNA contributes to global chromatin regulation. Proc Natl Acad Sci U S A 106: 17829–17834. doi: 10.1073/pnas.0906811106 19822756 10.1073/pnas.0906811106 PMC2764911

[17] Gibbons JG, Branco AT, Yu S, Lemos B (2014) Ribosomal DNA copy number is coupled with gene expression variation and mitochondrial abundance in humans. Nat Commun 5: 4850 doi: 10.1038/ncomms5850 25209200 10.1038/ncomms5850

[18] Larson K, Yan SJ, Tsurumi A, Liu J, Zhou J, et al (2012) Heterochromatin formation promotes longevity and represses ribosomal RNA synthesis. PLoS Genet 8: e1002473 doi: 10.1371/journal.pgen.1002473 22291607 10.1371/journal.pgen.1002473 PMC3266895

[19] Nemeth A, Conesa A, Santoyo-Lopez J, Medina I, Montaner D, et al (2010) Initial genomics of the human nucleolus. PLoS Genet 6: e1000889 doi: 10.1371/journal.pgen.1000889 20361057 10.1371/journal.pgen.1000889 PMC2845662

[20] van Koningsbruggen S, Gierlinski M, Schofield P, Martin D, Barton GJ, et al (2010) High-resolution whole-genome sequencing reveals that specific chromatin domains from most human chromosomes associate with nucleoli. Mol Biol Cell 21: 3735–3748. doi: 10.1091/mbc.E10-06-0508 20826608 10.1091/mbc.E10-06-0508 PMC2965689

[21] Ide S, Miyazaki T, Maki H, Kobayashi T (2010) Abundance of ribosomal RNA gene copies maintains genome integrity. Science 327: 693–696. doi: 10.1126/science.1179044 20133573 10.1126/science.1179044

[22] Weinmann R (1972) Regulation of ribosomal RNA and 5s RNA synthesis in Drosophila melanogaster. I. Bobbed mutants. Genetics 72: 267–276. 4630583 10.1093/genetics/72.2.267 PMC1212826

[23] Zhou J, Sackton TB, Martinsen L, Lemos B, Eickbush TH, et al (2012) Y chromosome mediates ribosomal DNA silencing and modulates the chromatin state in Drosophila. Proc Natl Acad Sci U S A 109: 9941–9946. doi: 10.1073/pnas.1207367109 22665801 10.1073/pnas.1207367109 PMC3382510

[24] Holmfeldt L, Wei L, Diaz-Flores E, Walsh M, Zhang J, et al (2013) The genomic landscape of hypodiploid acute lymphoblastic leukemia. Nat Genet 45: 242–252. doi: 10.1038/ng.2532 23334668 10.1038/ng.2532 PMC3919793

[25] Robinson G, Parker M, Kranenburg TA, Lu C, Chen X, et al (2012) Novel mutations target distinct subgroups of medulloblastoma. Nature 488: 43–48. doi: 10.1038/nature11213 22722829 10.1038/nature11213 PMC3412905

[26] Berger MF, Lawrence MS, Demichelis F, Drier Y, Cibulskis K, et al (2011) The genomic complexity of primary human prostate cancer. Nature 470: 214–220. doi: 10.1038/nature09744 21307934 10.1038/nature09744 PMC3075885

[27] Francis JM, Kiezun A, Ramos AH, Serra S, Pedamallu CS, et al (2013) Somatic mutation of CDKN1B in small intestine neuroendocrine tumors. Nat Genet 45: 1483–1486. doi: 10.1038/ng.2821 24185511 10.1038/ng.2821 PMC4239432

[28] Perry JA, Kiezun A, Tonzi P, Van Allen EM, Carter SL, et al (2014) Complementary genomic approaches highlight the PI3K/mTOR pathway as a common vulnerability in osteosarcoma. Proc Natl Acad Sci U S A 111: E5564–5573. doi: 10.1073/pnas.1419260111 25512523 10.1073/pnas.1419260111 PMC4280630

[29] Dulak AM, Stojanov P, Peng S, Lawrence MS, Fox C, et al (2013) Exome and whole-genome sequencing of esophageal adenocarcinoma identifies recurrent driver events and mutational complexity. Nat Genet 45: 478–486. doi: 10.1038/ng.2591 23525077 10.1038/ng.2591 PMC3678719

[30] James SA, West C, Davey RP, Dicks J, Roberts IN (2016) Prevalence and Dynamics of Ribosomal DNA Micro-heterogeneity Are Linked to Population History in Two Contrasting Yeast Species. Sci Rep 6: 28555 doi: 10.1038/srep28555 27345953 10.1038/srep28555 PMC4921842

[31] El-Salem M, Raghunath PN, Marzec M, Liu X, Kasprzycka M, et al (2009) Activation of mTORC1 signaling pathway in AIDS-related lymphomas. Am J Pathol 175: 817–824. doi: 10.2353/ajpath.2009.080451 19608873 10.2353/ajpath.2009.080451 PMC2716976

[32] Honjo S, Ajani JA, Scott AW, Chen Q, Skinner HD, et al (2014) Metformin sensitizes chemotherapy by targeting cancer stem cells and the mTOR pathway in esophageal cancer. Int J Oncol 45: 567–574. doi: 10.3892/ijo.2014.2450 24859412 10.3892/ijo.2014.2450 PMC4091970

[33] Tasioudi KE, Sakellariou S, Levidou G, Theodorou D, Michalopoulos NV, et al (2015) Immunohistochemical and molecular analysis of PI3K/AKT/mTOR pathway in esophageal carcinoma. APMIS 123: 639–647. doi: 10.1111/apm.12398 25912437 10.1111/apm.12398

[34] Zhu J, Wang M, Zhu M, He J, Wang JC, et al (2015) Associations of PI3KR1 and mTOR polymorphisms with esophageal squamous cell carcinoma risk and gene-environment interactions in Eastern Chinese populations. Sci Rep 5: 8250 doi: 10.1038/srep08250 25654238 10.1038/srep08250 PMC4318264

[35] Yin Y, Shen WH (2008) PTEN: a new guardian of the genome. Oncogene 27: 5443–5453. doi: 10.1038/onc.2008.241 18794879 10.1038/onc.2008.241

[36] Perry JM, He XC, Sugimura R, Grindley JC, Haug JS, et al (2011) Cooperation between both Wnt/{beta}-catenin and PTEN/PI3K/Akt signaling promotes primitive hematopoietic stem cell self-renewal and expansion. Genes Dev 25: 1928–1942. doi: 10.1101/gad.17421911 21890648 10.1101/gad.17421911 PMC3185965

[37] Zhang J, Grindley JC, Yin T, Jayasinghe S, He XC, et al (2006) PTEN maintains haematopoietic stem cells and acts in lineage choice and leukaemia prevention. Nature 441: 518–522. doi: 10.1038/nature04747 16633340 10.1038/nature04747

[38] Guo W, Lasky JL, Chang CJ, Mosessian S, Lewis X, et al (2008) Multi-genetic events collaboratively contribute to Pten-null leukaemia stem-cell formation. Nature 453: 529–533. doi: 10.1038/nature06933 18463637 10.1038/nature06933 PMC2840044

[39] Shen WH, Balajee AS, Wang J, Wu H, Eng C, et al (2007) Essential role for nuclear PTEN in maintaining chromosomal integrity. Cell 128: 157–170. doi: 10.1016/j.cell.2006.11.042 17218262 10.1016/j.cell.2006.11.042

[40] Fraser M, Zhao H, Luoto KR, Lundin C, Coackley C, et al (2012) PTEN deletion in prostate cancer cells does not associate with loss of RAD51 function: implications for radiotherapy and chemotherapy. Clin Cancer Res 18: 1015–1027. doi: 10.1158/1078-0432.CCR-11-2189 22114138 10.1158/1078-0432.CCR-11-2189 PMC3378487

[41] Bassi C, Ho J, Srikumar T, Dowling RJ, Gorrini C, et al (2013) Nuclear PTEN controls DNA repair and sensitivity to genotoxic stress. Science 341: 395–399. doi: 10.1126/science.1236188 23888040 10.1126/science.1236188 PMC5087104

[42] Zhang C, Comai L, Johnson DL (2005) PTEN represses RNA Polymerase I transcription by disrupting the SL1 complex. Mol Cell Biol 25: 6899–6911. doi: 10.1128/MCB.25.16.6899-6911.2005 16055704 10.1128/MCB.25.16.6899-6911.2005 PMC1190253

[43] Woiwode A, Johnson SA, Zhong S, Zhang C, Roeder RG, et al (2008) PTEN represses RNA polymerase III-dependent transcription by targeting the TFIIIB complex. Mol Cell Biol 28: 4204–4214. doi: 10.1128/MCB.01912-07 18391023 10.1128/MCB.01912-07 PMC2423115

[44] Signer RA, Magee JA, Salic A, Morrison SJ (2014) Haematopoietic stem cells require a highly regulated protein synthesis rate. Nature 509: 49–54. doi: 10.1038/nature13035 24670665 10.1038/nature13035 PMC4015626

[45] Kobayashi T, Ganley AR (2005) Recombination regulation by transcription-induced cohesin dissociation in rDNA repeats. Science 309: 1581–1584. doi: 10.1126/science.1116102 16141077 10.1126/science.1116102

[46] Zentner GE, Balow SA, Scacheri PC (2014) Genomic characterization of the mouse ribosomal DNA locus. G3 (Bethesda) 4: 243–254. 24347625 10.1534/g3.113.009290 PMC3931559

[47] Grandori C, Gomez-Roman N, Felton-Edkins ZA, Ngouenet C, Galloway DA, et al (2005) c-Myc binds to human ribosomal DNA and stimulates transcription of rRNA genes by RNA polymerase I. Nat Cell Biol 7: 311–318. doi: 10.1038/ncb1224 15723054 10.1038/ncb1224

[48] Dominguez-Sola D, Ying CY, Grandori C, Ruggiero L, Chen B, et al (2007) Non-transcriptional control of DNA replication by c-Myc. Nature 448: 445–451. doi: 10.1038/nature05953 17597761 10.1038/nature05953

[49] Zentner GE, Saiakhova A, Manaenkov P, Adams MD, Scacheri PC (2011) Integrative genomic analysis of human ribosomal DNA. Nucleic Acids Res 39: 4949–4960. doi: 10.1093/nar/gkq1326 21355038 10.1093/nar/gkq1326 PMC3130253

[50] Pontvianne F, Carpentier MC, Durut N, Pavlistova V, Jaske K, et al (2016) Identification of Nucleolus-Associated Chromatin Domains Reveals a Role for the Nucleolus in 3D Organization of the A. thaliana Genome. Cell Rep 16: 1574–1587. doi: 10.1016/j.celrep.2016.07.016 27477271 10.1016/j.celrep.2016.07.016 PMC5279810

[51] Feng J, Liang J, Li J, Li Y, Liang H, et al (2015) PTEN Controls the DNA Replication Process through MCM2 in Response to Replicative Stress. Cell Rep 13: 1295–1303. doi: 10.1016/j.celrep.2015.10.016 26549452 10.1016/j.celrep.2015.10.016

[52] Flach J, Bakker ST, Mohrin M, Conroy PC, Pietras EM, et al (2014) Replication stress is a potent driver of functional decline in ageing haematopoietic stem cells. Nature 512: 198–202. doi: 10.1038/nature13619 25079315 10.1038/nature13619 PMC4456040

[53] Rusiniak ME, Kunnev D, Freeland A, Cady GK, Pruitt SC (2012) Mcm2 deficiency results in short deletions allowing high resolution identification of genes contributing to lymphoblastic lymphoma. Oncogene 31: 4034–4044. doi: 10.1038/onc.2011.566 22158038 10.1038/onc.2011.566 PMC3309111

[54] Truitt ML, Ruggero D (2016) New frontiers in translational control of the cancer genome. Nat Rev Cancer 16: 288–304. doi: 10.1038/nrc.2016.27 27112207 10.1038/nrc.2016.27 PMC5491099

[55] Xu B, Lee KK, Zhang L, Gerton JL (2013) Stimulation of mTORC1 with L-leucine rescues defects associated with Roberts syndrome. PLoS Genet 9: e1003857 doi: 10.1371/journal.pgen.1003857 24098154 10.1371/journal.pgen.1003857 PMC3789817

[56] Bose T, Lee KK, Lu S, Xu B, Harris B, Slaughter B, Unruh J, Garrett A, McDowell W, Box A, Li H, Peak A, Ramachandran S, Seidel C, Gerton JL (2012) Cohesin proteins promote ribosomal RNA production and protein translation in yeast and human cells. PLoS Genet 8: e1002749 https://doi.org/10.1371/journal.pgen.1002749 22719263 10.1371/journal.pgen.1002749 PMC3375231

