# Supplementary figures and images for: Ribosomal DNA copy number loss and sequence variation in cancer
Source: PLoS Genet. 2017 Jun 22;13(6):e1006771. doi: 10.1371/journal.pgen.1006771 (PMC5480814; doi:10.1371/journal.pgen.1006771)

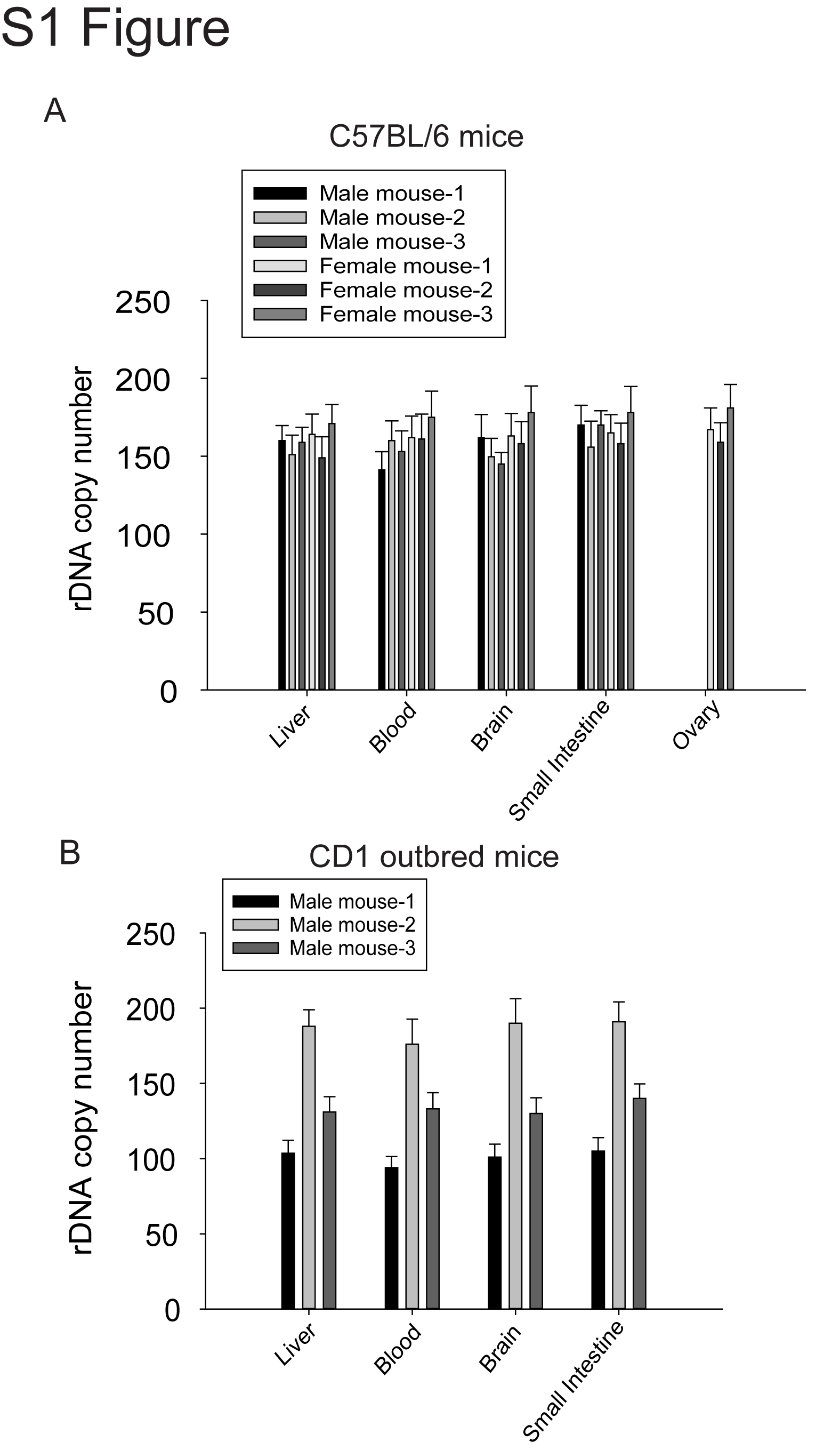

Supplement: S1 Fig — (A). The rDNA copy numbers are similar in four tissues from three C57BL/6 mice, (B). The rDNA copy numbers are similar in four tissues from three CD1 mice, but have significant differences between individuals in the outbred strain. (TIF) [file pgen.1006771.s001.tif]

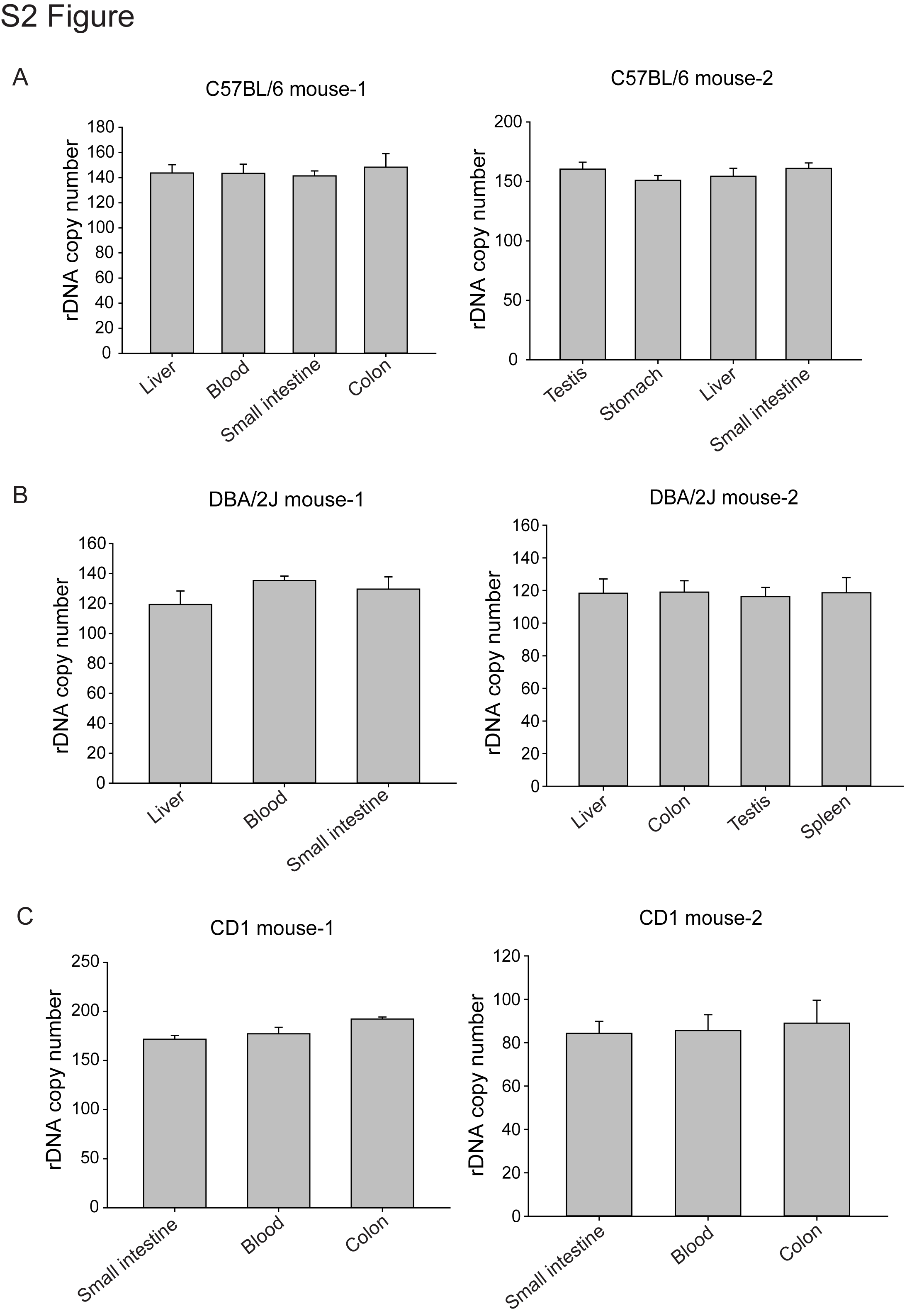

Supplement: S2 Fig — (A). The rDNA copy number is similar in different portions of four tissues from two C57BL/6 mice. (B). The rDNA copy number is similar in different portions of tissues from two DBA/2J mice. (C). The rDNA copy number is similar in different portions of three tissues from two CD1 mice. (TIF) [file pgen.1006771.s002.tif]

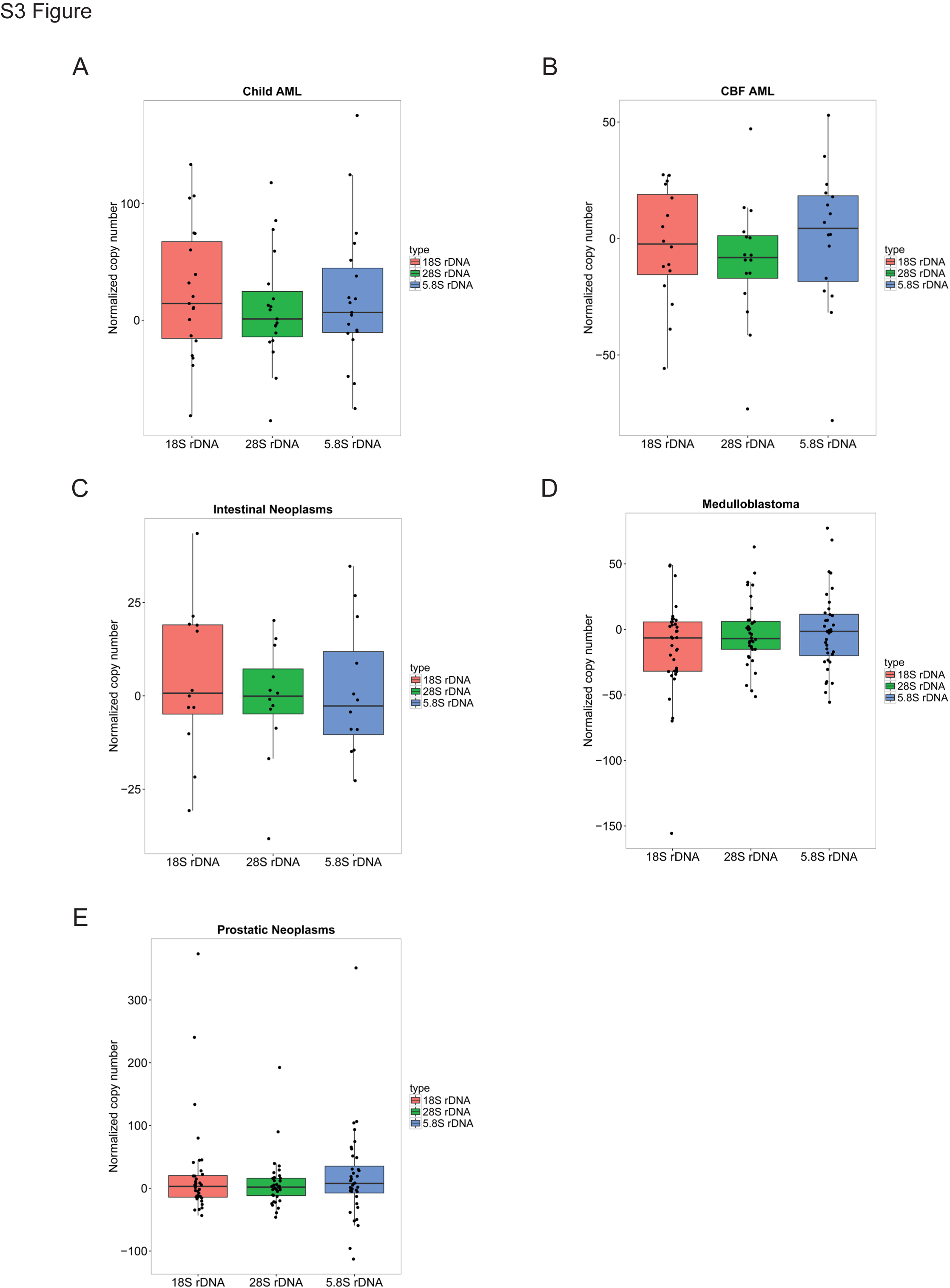

Supplement: S3 Fig — (A) Child Acute lymphoblastic leukemia, (B) Core binding factor acute myeloid leukemia, (C) Intestinal Neoplasms (Liver/Small bowel), (D) Medulloblastoma, (E) Prostatic neoplasms. (TIF) [file pgen.1006771.s003.tif]

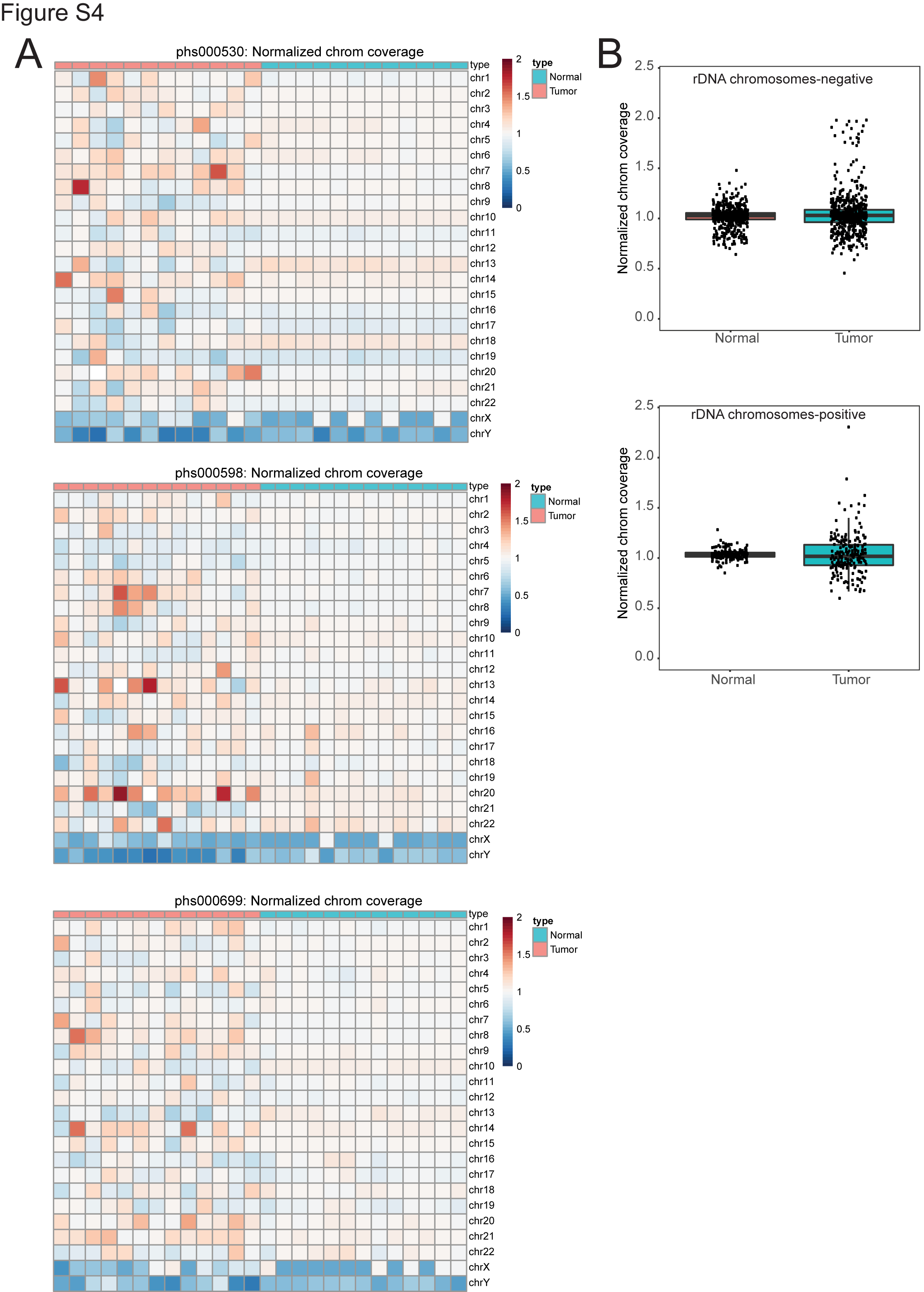

Supplement: S4 Fig — A. Normalized coverage of each chromosome for tumor (left) and normal (right) genome for each person is shown in a heatmap for each of the 3 projects for which we calculated loss of copies of rDNA. The tumor genomes show more variable coverage than the normal genomes, indicating aneuploidy, as might be expected. B. For the 5 human chromosomes containing rDNA (13, 14, 15, 21, 22), the normalized coverage is plotted for tumor and normal genomes for all genome projects negative for loss of rDNA (rDNA chromosomes-negative) and positive for loss of rDNA (rDNA chromosomes-positive). While there is clearly more variable coverage in the tumor genomes for these chromosomes in both positive and negative genome projects, there is not a trend toward coverage loss in the positive projects that could account for the loss of rDNA. (TIF) [file pgen.1006771.s004.tif]

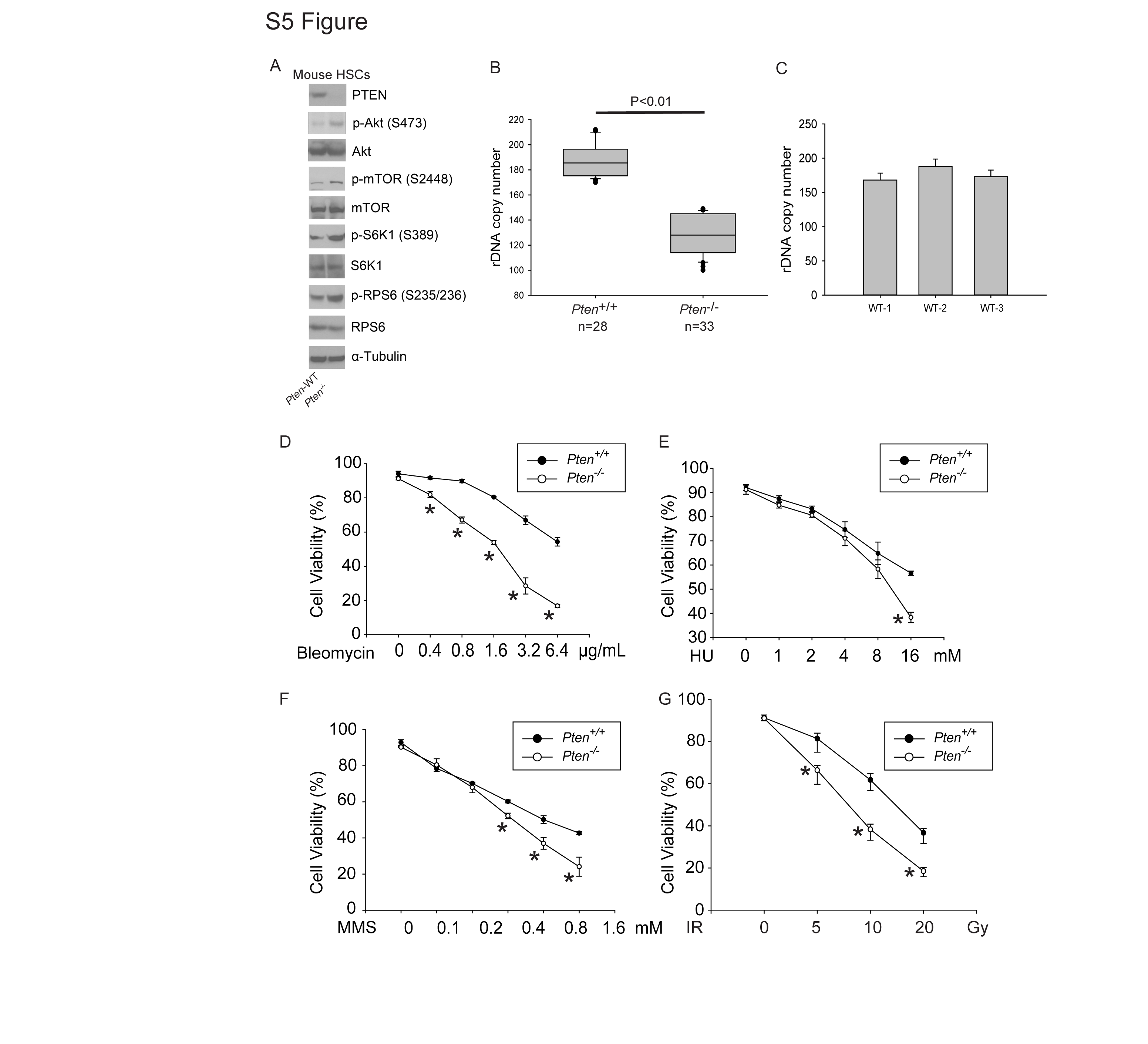

Supplement: S5 Fig — (A) Western blotting for various indicators of mTOR activity was performed to confirm that loss of PTEN results in activation of mTOR activity in HSCs. (B) Pten-/- HSCs exhibited a decrease in rDNA copy number compared to matched WT HSCs, samples were derived from 3 mice of each genotype with 8–12 clones each (n = 28 for Pten+/+ HSCs, and n = 33 for Pten-/- HSCs). (C). The rDNA copy number in the gDNA derived from tail samples was similar between the WT mice used to derive the HSC clones used in (B), and was also similar to rDNA copy number in Pten+/+ HSC clones derived from these mice. Tail DNA was not available for the mice used to derive the HSC clones in (B). (D-G). HSCs were treated as indicated and the viability was calculated at day five based on total cell number and trypan blue staining. Each dosage was performed in triplicate and the error bars represent standard deviation. Data shown was derived from HSCs from a single mouse of each genotype. Asterisks represent values for which a t test indicates statistical significance below 0.05. (TIF) [file pgen.1006771.s005.tif]

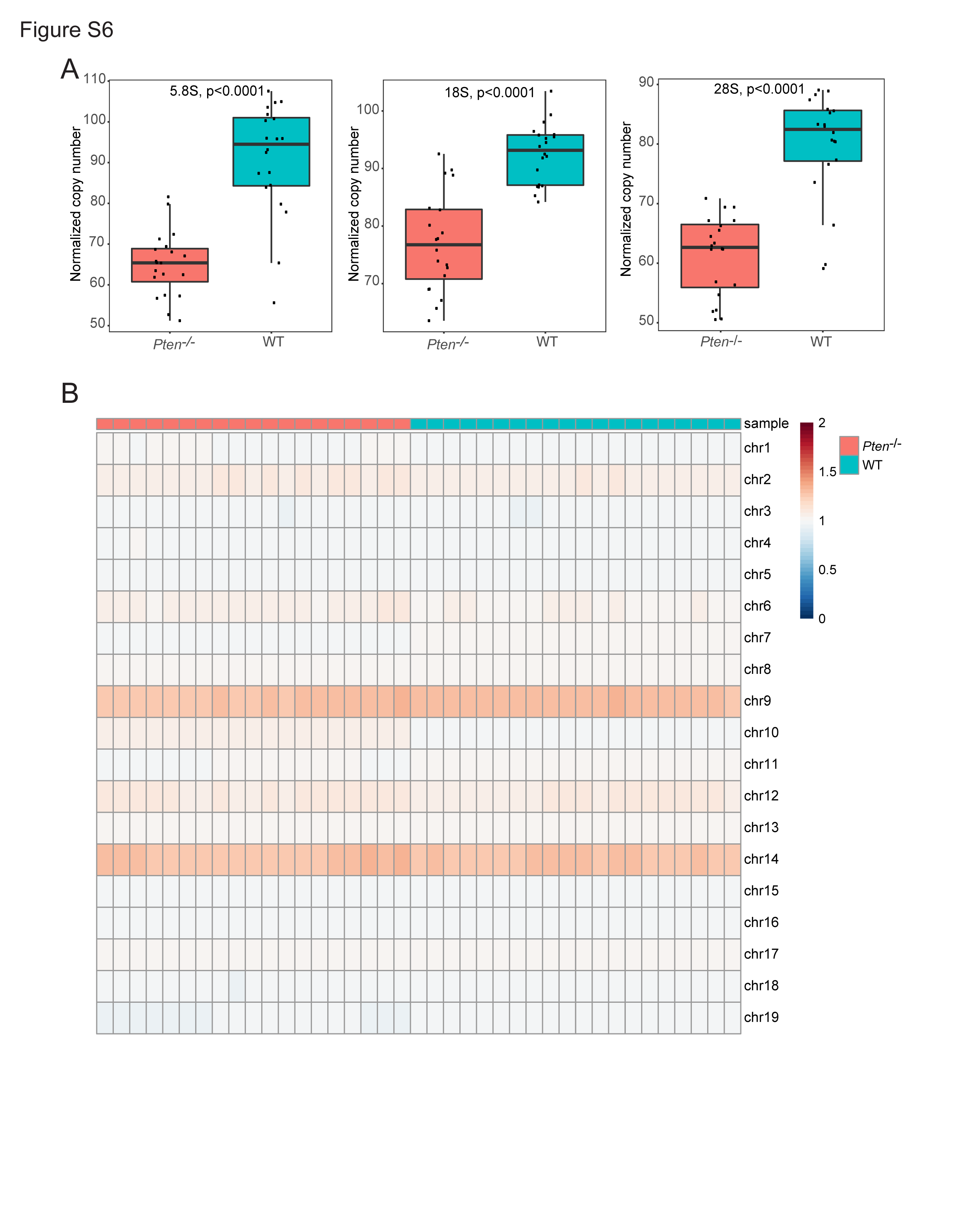

Supplement: S6 Fig — A. DNA was isolated from 20 WT and 20 Pten-/- HSC clones. Libraries were made using the Nextera kit and subjected to sequencing at low coverage. We employed our computational pipeline developed for the human cancer genome data to analyze these samples. (A) We found a loss of 20–30 copies of rDNA in the Pten-/- clones, depending on the sequence counted (5.8S, 18S, or 28S). For each of the 3 sequences, a t test showed statistical significance lower than 0.0001. (B) ~16,000 single copy exons were used to examine ploidy. Coverage is plotted as a heat map by the average coverage for each chromosome for each HSC clone, with the genotype indicated. There are no obvious differences in chromosome coverage between the WT and Pten-/- clones, indicating that the WT and Pten-/- clones are similarly euploid. (TIF) [file pgen.1006771.s006.tif]

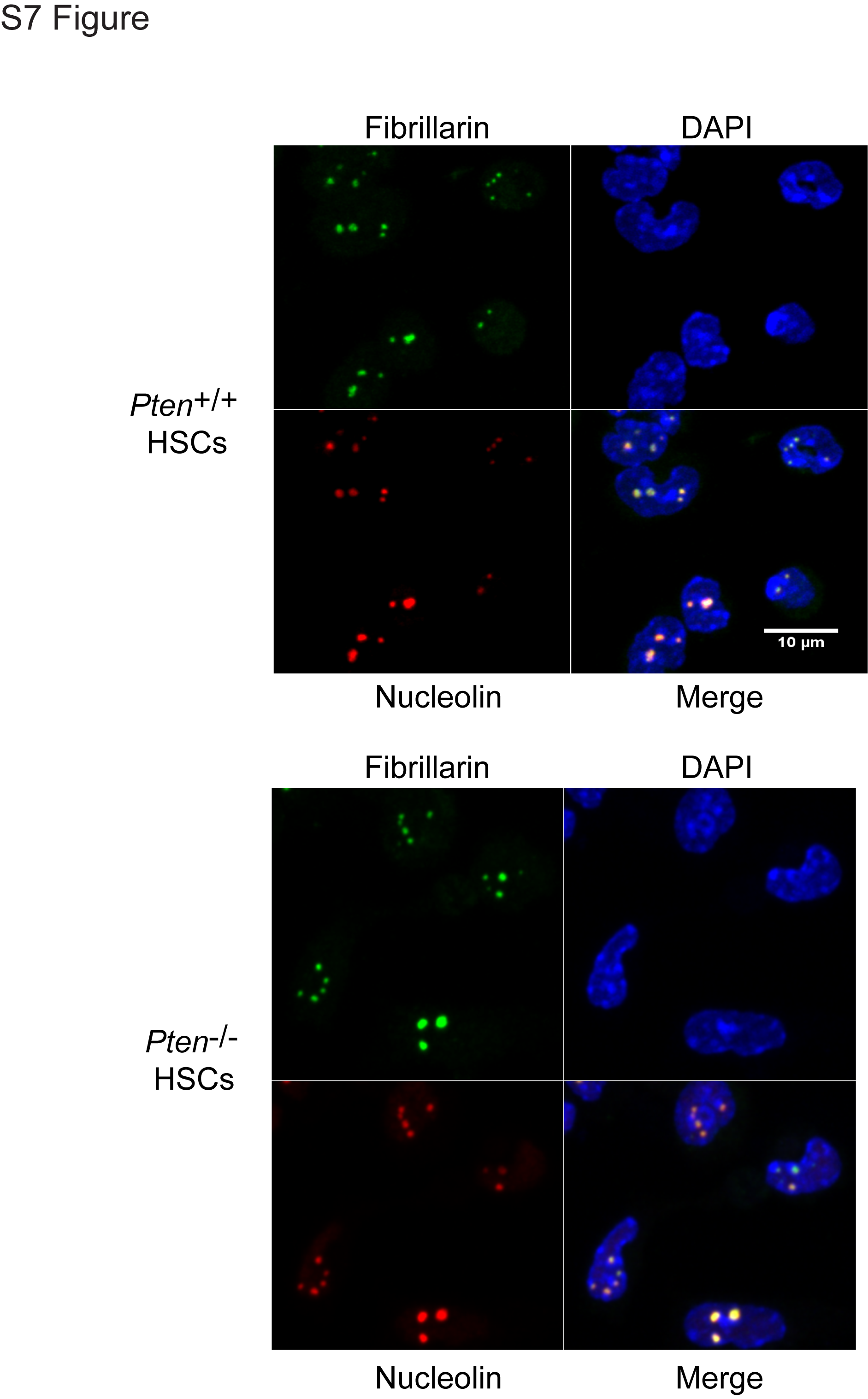

Supplement: S7 Fig — (TIF) [file pgen.1006771.s007.tif]

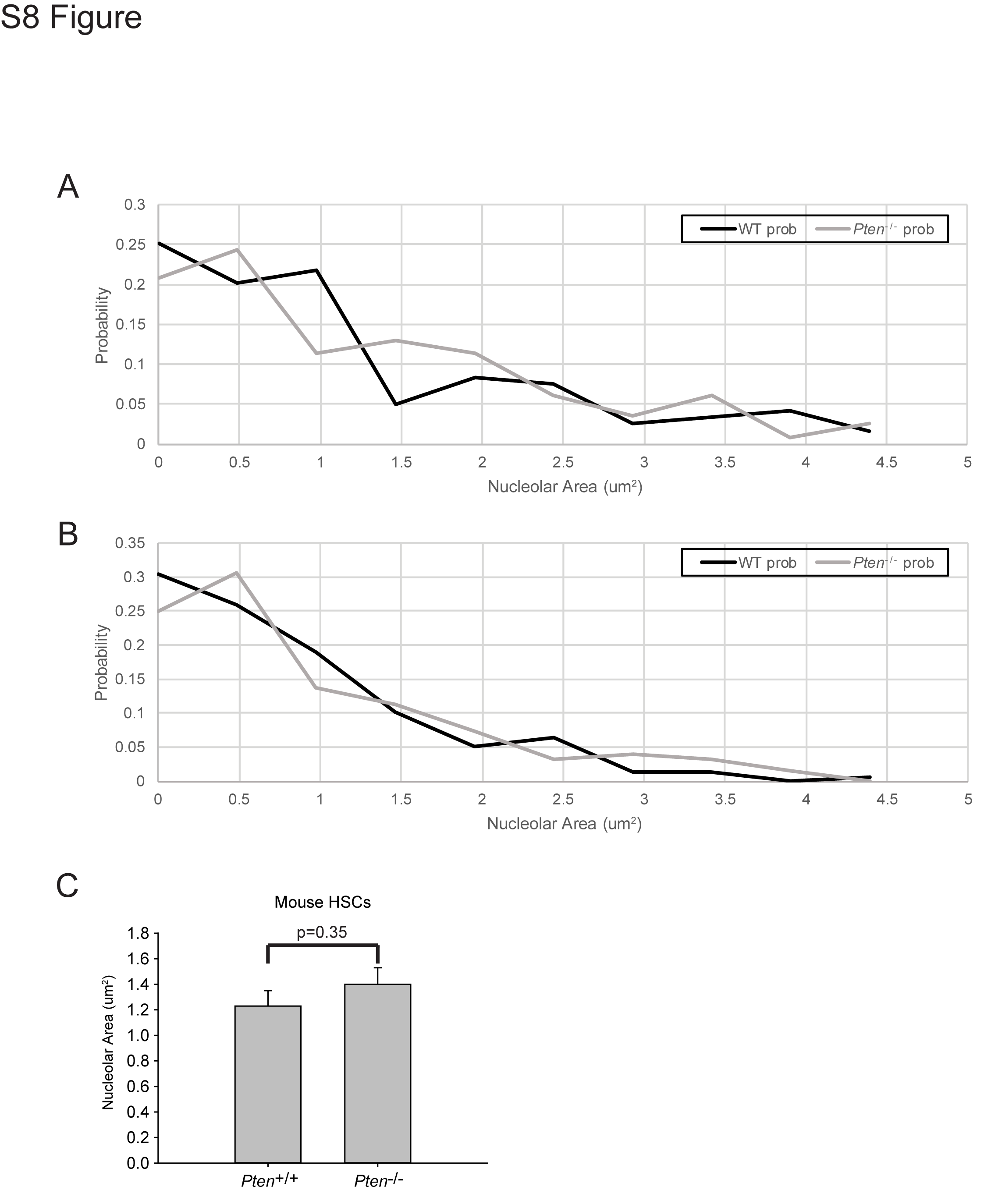

Supplement: S8 Fig — Nucleolar size measurements of the immunofluorescence staining of nucleolin (A) and fibrillarin (B) in WT and Pten-/- HSCs. The average density distribution of nucleolar area is plotted. (C) Average area of fibrillarin staining was quantified and compared. About 40 cells were quantified for each sample with seven replicates. (TIF) [file pgen.1006771.s008.tif]

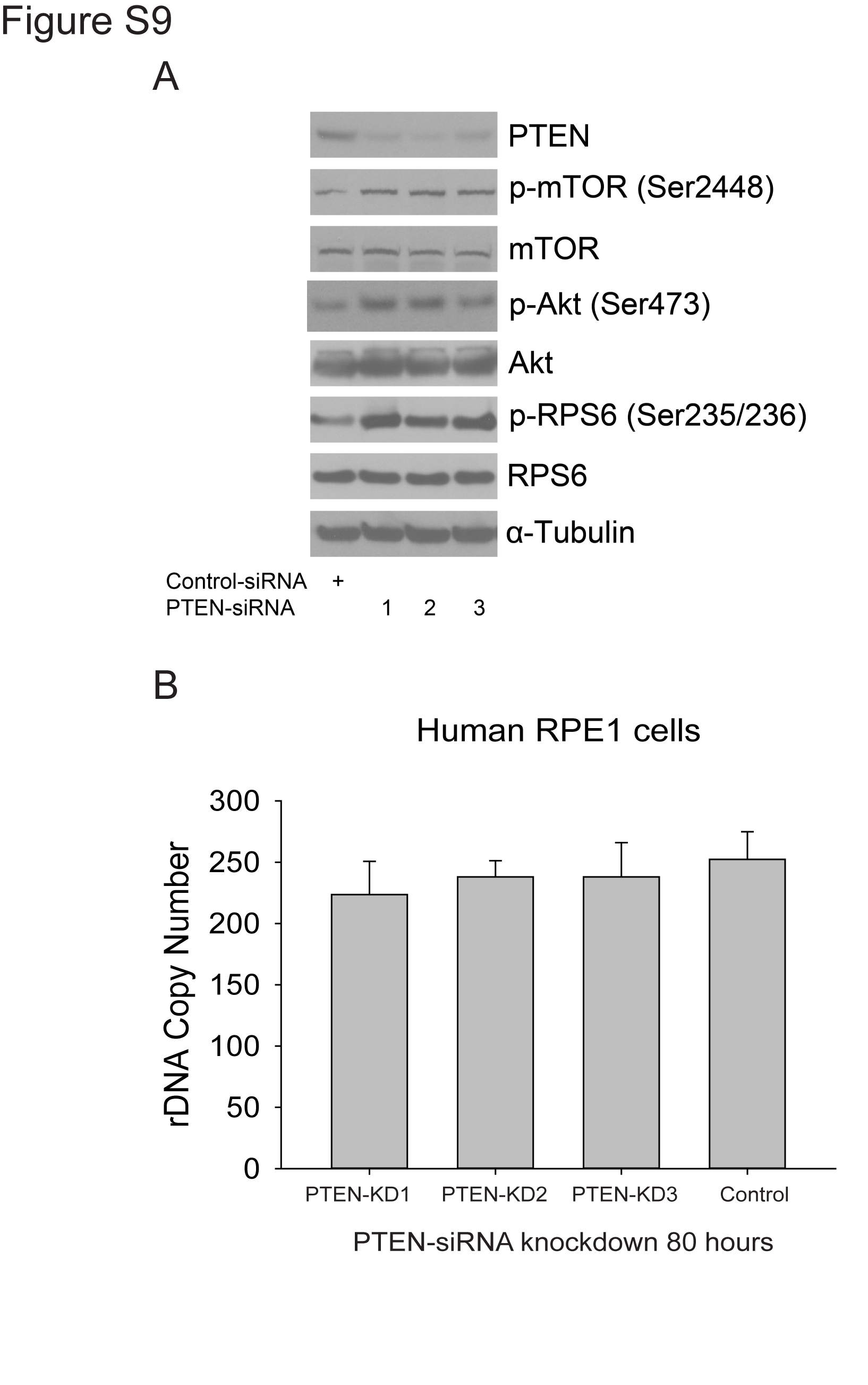

Supplement: S9 Fig — A. RPE cells were transfected with three different siRNAs to PTEN. The efficiency of knockdown was monitored by Western blot after 80 hours. Other proteins in the mTOR pathway were also monitored by Western blot. B. ddPCR was performed on genomic DNA isolated from cultures 80 hours post-transfection. ddPCR was performed for the human 45S gene with TBP as the single copy reference gene. Error bars represent the standard deviation of triplicate reactions. (TIF) [file pgen.1006771.s009.tif]
